# Designing AAV Vectors for Monitoring the Subtle Calcium Fluctuations of Inferior Olive Network *in vivo*

**DOI:** 10.3389/fncel.2022.825056

**Published:** 2022-04-27

**Authors:** Kevin Dorgans, Da Guo, Kiyoto Kurima, Jeff Wickens, Marylka Yoe Uusisaari

**Affiliations:** ^1^Neuronal Rhythms in Movement Unit, Okinawa Institute of Science and Technology Graduate University, Okinawa, Japan; ^2^Neurobiology Research Unit, Okinawa Institute of Science and Technology Graduate University, Okinawa, Japan

**Keywords:** genetic targeting, adeno-associated virus (AAV), subthreshold oscillation, calcium imaging, cerebellum, inferior olive

## Abstract

Adeno-associated viral (AAV) vectors, used as vehicles for gene transfer into the brain, are a versatile and powerful tool of modern neuroscience that allow identifying specific neuronal populations, monitoring and modulating their activity. For consistent and reproducible results, the AAV vectors must be engineered so that they reliably and accurately target cell populations. Furthermore, transgene expression must be adjusted to sufficient and safe levels compatible with the physiology of studied cells. We undertook the effort to identify and validate an AAV vector that could be utilized for researching the inferior olivary (IO) nucleus, a structure gating critical timing-related signals to the cerebellum. By means of systematic construct generation and quantitative expression profiling, we succeeded in creating a viral tool for specific and strong transfection of the IO neurons without adverse effects on their physiology. The potential of these tools is demonstrated by expressing the calcium sensor GCaMP6s in adult mouse IO neurons. We could monitor subtle calcium fluctuations underlying two signatures of intrinsic IO activity: the subthreshold oscillations (STOs) and the variable-duration action potential waveforms both *in-vitro* and *in-vivo*. Further, we show that the expression levels of GCaMP6s allowing such recordings are compatible with the delicate calcium-based dynamics of IO neurons, inviting future work into the network dynamics of the olivo-cerebellar system in behaving animals.

## 1. Introduction

There is no doubt that genetic modification methodology involving transgenic animals and viral vector-based techniques has transformed neuroscience in the last decades, allowing monitoring and modulating neuronal activity in unprecedented detail and breadth (Chen et al., 2005; Lu et al., 2016). Large-scale genetic screening and engineering efforts have resulted in numerous transgenic driver mouse lines that allow relatively easy targeting of genetic material (Daigle et al., 2018; Mehta et al., 2019), especially in the context of brain regions where neuronal subpopulations are well-identifiable based on molecular markers (Madisen et al., 2012; Luo et al., 2018). This general approach brings additional benefits in an era where ethical scientific work requires reducing experimental animals use, as it allows optimizing methodology for the specific brain regions or neuron types and better reproducibility. However, use of genetically modified animals is often impractical for smaller research teams as significant investment of resources is required for development and maintenance of transgenic mouse lines.

Even when not limited by resources, stereotactic delivery of a viral vectors (such as adeno-associated virus (AAV)-based) is often the safest and only option for localized targeting specific cell populations in less-thoroughly characterized brain nuclei, but the small packing capacity of AAV vectors limits the size of genetic sequences that can be delivered (Taniguchi et al., 2011; Samulski and Muzyczka, 2014). Thus, readily-available commercial vectors are often offered only with a few generic short-form promoters (<3.7 kb; e.g., such as synapsin (Syn), cytomegalovirus (CMV), Calcium/calmodulin-dependent protein kinase II (CaMKII), Elongation factor 1α (EF1) and the synthetic promoter CAG that can be used with larger transgenes (such as genetically-encoded activity reporters or optogenetic activators). Unfortunately, these short, “generic” constructs often have poor efficiency for targeting neurons outside of the cerebral cortex. The long-form (>3.7 kb) promoters utilized in driving specific, non-cortical gene expression in transgenic mouse lines are rarely compatible with AAV-vector applications, and it is thus necessary to screen shorter (<3.7 kb) clones of the promoters for selective targeting of each brain region of interest.

Among the brain structures where research has suffered from such lack of genetic targeting tools is the inferior olive (IO), a brain structure that gates critical timing-related signals to the cerebellum (Devor, 2002; De Gruijl et al., 2013; Streng et al., 2018). The IO neurons are characterized by two unusual electrophysiological features that are presumed to play decisive roles in the timing functions of the entire olivo-cerebellar system: the subthreshold membrane voltage oscillations (STOs) and a variable-duration action potential with a prominent “calcium shoulder” (Llinás and Yarom, 1981; Benardo and Foster, 1986; Chorev et al., 2007a; Mathy et al., 2009). The involvement of various voltage-gated calcium currents distributed in somatic and dendritic compartments in driving and modulating both STOs and spike width variations has been described decades ago, but the underlying mechanisms remain poorly understood. Previously, voltage imaging methodology has been used in the context of IO STOs (see Leznik and Llinás (2005); Devor and Yarom (2002); Dorgans et al. (2020)), but only calcium imaging would provide the necessary spatiotemporal information on these intracellular processes. On the other hand, the STOs and spike width variations are likely to be exquisitely sensitive to perturbations of calcium buffering (Rekling et al., 2012; Yu et al., 2014 (Iwaniuk et al., 2009)), raising concerns that calcium probe presence would lead to aberrant or completely absent STOs or spike-width variations and therefore disqualify calcium imaging methodology entirely from behavioral use.

Despite several works having employed a generic targeting approach for IO (White and Sillitoe, 2017; Rowan et al., 2018; Gaffield et al., 2019; González-Calvo et al., 2021), no systematic effort has been made to create a more specific targeting tool. Instead, experimenters have resorted to indirect methods, such as imaging IO-axon-evoked spikes in the cerebellum (Ju et al., 2019; Hoang et al., 2020; Roh et al., 2020; Michikawa et al., 2021), optogenetic modulation of IO afferents instead of IO neurons (Kim et al., 2020), limiting experimentation to cerebellar cortical regions where off-target transfection of axons with, e.g., the CamKII promoter is not problematic (Mathews et al., 2012; Gaffield et al., 2019) or morphological analysis (Nishiyama et al., 2007; Pätz et al., 2018; Vrieler et al., 2019; González-Calvo et al., 2021). To our knowledge, there have been no published reports of *in situ, in vivo* multi-cell IO network activity besides our recently-published preliminary observations (Guo et al., 2021).

It is not clear why targeted viral expression in the IO with generic promoters has been so challenging. Nevertheless, it is highly likely that high viral titers, long transfection times and using strong unspecific and/or artificial promoters may lead to pathological changes (Miyashita et al., 2013) and induce dysfunction in the targeted network (Miyashita et al., 2013; Steinmetz et al., 2017; Kim et al., 2020). Even if no dramatic pathological changes was observed, overloading a brain structure with viral particles can lead to unspecific or trans-synaptic expression patterns and, thus, unreliable experimental results. Notably, while in our previous report (Guo et al., 2021) we were able to record IO neuron activity using a commercial CAG-promoter-driven GCaMP6s-expression vector, the expression efficacy and neuron-specificity was highly unreliable, leading to initiation of the present work.

Here, we describe the identification of a novel genetic targeting tool that induces specific and strong transgene expression in the IO with low viral titers (1.10^11^vg/ml) and short expression periods. For this purpose, we constructed and screened a number of candidate promoter sequences for their suitability for driving expression of a high-affinity genetically encoded calcium indicator GCaMP6s (Chen et al., 2013) in combination with three different viral capsids (AAV9, AAV-PHP.S, and AAV-PHP.eB; (Chan et al., 2017; Mathiesen et al., 2020; Maturana et al., 2021)) and the tetracycline-transactivator (tTA) / tetracycline response element (TRE) expression enhancer system (Gossen and Bujard, 1992). The best constructs utilize a 3.7kb clone of serotonin receptor subtype 5b [Htr5b(3.7)] promoter sequence. Using these AAV constructs compatible with the physiology of IO neurons, we show feasibility of (1) expressing calcium probes in IO without abolishing STOs and spike shape variability and (2) monitoring the STOs, features of IO spikes and climbing fiber activity using 1-photon GCaMP6s imaging both *in-vitro* and *in-vivo*.

## 2. Methods

### 2.1. Production of Custom AAV-Vectors

The GCaMP6s reporter plasmid was prepared from an AAV plasmid with GCaMP6s (Addgene #50942). The EGFP reporter was prepared from the GCaMP6s reporter plasmid by replacing GCaMP6s with EGFP. Because the size of genetic material that can be stably packaged in standard AAV constructions is limited to approximately 4.8 kb, we first removed the WPRE fragments and cloned approximately 3.7 kb promoter fragments. Shorter promoters were also made from some longer gene versions (Table 1) and the viruses were prepared with different serotypes (AAV9, AAV.PHP.S, or AAV.PHP.eB). Mouse genomic DNA was used to amplify the upstream regions of genes, using primers listed in Table 1 and cloned into the reporter plasmids expressing EGFP (for anatomical study) or GCaMP6s (for functional study). Two categories of AAVs were generated with 3 different capsids (AAV9, AAV.PHP.S, AAV.PHP.eB).

1. “single-AAVs” where transgene is under the control of the promoters studied (Table 1).
2. “dual-AAV,” where the transgene is indirectly under the control of the promoter through tTA/TRE-enhanced expression systems. (Chtarto et al., 2003). Plasmids with tTA (tetracycline-controlled transactivator) and TRE (Tet Response Element) were prepared from AAV plasmids Addgene #104109 and #104110, respectively (Table 3).

**Table 1:** List of custom AAV vectors used for anatomical and functional characterization of IO by “single-AAV” and “double-AAV” approaches.

**Construct**	**Gene**	**Primers to amplify promoter region**	**Serotype**	**Titer (vg/ml)**
pAAV-CAG-tdTomato	Addgene#59462	—	PHP.S	3.10^12^
pAAV-CAG-EGFP	Addgene#59462	*tdTomato is replaced with EGFP*	AAV9	3.10^13^
			PHP.eB	1,7.10^13^
pAAV-Igsf (3.7)-EGFP	MGI:2135283	*Forward*: GGATGATCACCCTGCCCTGGCATTCCCCTACACTAGGGCATTG *Reverse*: GATCGAATTCCTGCTCTGCACAGCACAGCTCTGCTCTCGCAG	AAV9	3.10^12^
pAAV-Igsf (2.5)-EGFP	MGI:2135283	*Forward*: GAGCCGTATTTCAATTCTACAAAGACC *Reverse*: GATCGAATTCCTGCTCTGCACAGCACAGCTCTGCTCTCGCAG	AAV9	4,3.10^12^
pAAV-Igsf (1.3)-EGFP	MGI:2135283	*Forward*: TCTAGACAATTTCACATGAGCCTGGTGGG *Reverse*: GATCGAATTCCTGCTCTGCACAGCACAGCTCTGCTCTCGCAG	AAV9	2,5.10^12^
pAAV-Htr5b (3.7)-EGFP	MGI:96284	*Forward*: ATGATGATCACATAACCACACTGAAGATCAGAGAAGAAC *Reverse*: ATGACAATTGGGGACTTGGGCTCTGGGGCAGGAGATGTGCC	AAV9	5.10^11^
pAAV-htr5b (3.7)-mTagBFP2	Addgene#100799	*Forward*: ATGATGATCACATAACCACACTGAAGATCAGAGAAGAAC *Reverse*: CTCGAACTCGAGTTAATTGAGCTTGTGCCCCAG	PHP.eB	1.1 × 1012
pAAV-Htr5b (3.0)-EGFP	MGI:96284	*Forward*: CATAACCACACTGAAGATCAGAGAAGAAC *Reverse*: ATGACAATTGGGGACTTGGGCTCTGGGGCAGGAGATGTGCC	AAV9	2.10^14^
pAAV-Htr5b (1.8)-EGFP	MGI:96284	*Forward*: ATGCATATTACTATGAATAGCTATGTAC *Reverse*: ATGACAATTGGGGACTTGGGCTCTGGGGCAGGAGATGTGCC	AAV9	5.10^13^
pAAV-Htr5b (1.0)-EGFP	MGI:96284	*Forward*: AAAGAATGAGATTGAAAATGGAATG *Reverse*: ATGACAATTGGGGACTTGGGCTCTGGGGCAGGAGATGTGCC	AAV9	3.10^14^
pAAV-Pdx1 (3.7)-EGFP	MGI:102851	*Forward*: ATGAGGATCCGTGTGCTTTCTTGATTGGCAGTTGTTGTGG *Reverse*: ATGACCATGGTGGCAGCCGGCACTTGGGGGCCAGC	AAV9	5.10^11^
pAAV-Susd4 (3.7)-EGFP	MGI:2138351	*Forward*: ATGAAGATCTCCATTATTTATAAACTATGTGG *Reverse*: ATGACAATTGGCAAGCGAGCGAGCGAGCAAGACAGAGGAGC	AAV9	6.10^11^
pAAV-Igsf (2.5)-GCaMP6s	MGI:2135283	*Forward*: GAGCCGTATTTCAATTCTACAAAGACC *Reverse*: GATCGAATTCCTGCTCTGCACAGCACAGCTCTGCTCTCGCAG	AAV9	3.10^12^
pAAV-Igsf (1.3)-GCaMP6s	MGI:2135283	*Forward*: TCTAGACAATTTCACATGAGCCTGGTGGG *Reverse*: GATCGAATTCCTGCTCTGCACAGCACAGCTCTGCTCTCGCAG	AAV9	2,5.10^12^
pAAV-Htr5b (3.0)-GCaMP6s	MGI:96284	*Forward*: CATAACCACACTGAAGATCAGAGAAGAAC *Reverse*: ATGACAATTGGGGACTTGGGCTCTGGGGCAGGAGATGTGCC	AAV9	3.10^14^
pAAV-Htr5b (1.8)-GCaMP6s	MGI:96284	*Forward*: CATAACCACACTGAAGATCAGAGAAGAAC *Reverse*: ATGACAATTGGGGACTTGGGCTCTGGGGCAGGAGATGTGCC	AAV9	5.10^11^
pAAV-Susd4 (2.5)-GCaMP6s	MGI:2138351	*Forward*: AGATCTAACATTGAGCATAACTTAG *Reverse*: ATGACAATTGGCAAGCGAGCGAGCGAGCAAGACAGAGGAGC	PHP.eB	4.10^12^
pAAV-Htr5b (3.7)-tTA	MGI:96284	*Forward*: ATGATGATCACATAACCACACTGAAGATCAGAGAAGAAC *Reverse*: ATGACAATTGGGGACTTGGGCTCTGGGGCAGGAGATGTGCC	AAV9	3.10^12^
			PHP.S	1.10^12^
			PHP.eB	5.10^11^
pAAV-TRE-EGFP	Addgene#89875	—	PHP.eB	3.10^11^
pAAV-TRE-GCaMP6s	Addgene#89875	*EGFP was replaced with GCaMP6s*	AAV9	1.10^13^
			PHP.S	1.10^13^
			PHP.eB	1.10^12^

Production and purification of AAV vectors was performed as follows:

1. HEK293T cells were seeded at 2 million cells per 100mm plate in Dulbecco's modified Eagle's medium (ThermoFisher Scientific, Minato, JP) containing 10% fetal bovine serum (FBS) 24 h prior to the transfection.
2. AAV from 3 serotypes, AAV9, AAV.PHP.S, AAV.PHP.eB (see Table 1 for details), helper, and expression plasmids were co-transfected by calcium phosphate transfection method (Jordan et al., 1996).
3. 6 h after the transfection, cells were washed with PBS, and incubated in medium containing 2% FBS.
4. 64 h after medium replacement cells containing virus particles were collected. After extraction by four cycles of freeze-and-thaw, the virus particles were purified from the crude lysate by three rounds of centrifugation.

### 2.2. Animal Ethics Statement

All animal experiments were performed in accordance with guidelines approved by the Okinawa Institute of Science and Technology Graduate University Institutional Animal Care and Use Committee (IACUC) in an Association for Assessment and Accreditation of Laboratory Animal Care (AAALAC International) accredited facility.

### 2.3. Intra-Cerebral Injection of Viral Particles Into the Inferior Olive

We have previously described in detail the procedures for precise stereotactic targeting of the IO (Dorgans et al., 2020; Guo et al., 2021). In brief, postnatal (P) 35-45 C57BL/6J male mice (CLEA Japan, Shizuoka, Japan) were anesthetized with 5% isoflurane (SomnoSuite, Kent Scientific, CT, USA) and maintained on a heat pad (38°*C*; TMP-5b, Supertech Instruments) attached to a stereotaxic frame (Neurostar, Tübingen, Germany) with constant 1% to 2.4% isoflurane delivery *via* a nose cone. Special attention is required to correctly align the head and body for reliable targeting of deep structures such as IO (Dorgans et al., 2020; Guo et al., 2021). After shaving the scalp, skin was locally anesthetized by application of Xylocaine gel (Xylogel, gel 2%, Aspen, Japan), disinfected and incised with a sharp blade to expose the skull, and small (about 1 mm diameter) craniotomies were opened with a hand-held drill (Surgic XT Plus drill, NSK Dental, Japan).

The purified virus solution (see titers in Table 1) was diluted in saline (pH 7.4, dilution ratio (depending on the stock titer) up to 1/500) to reach the target titer of (1.10^11^vg/ml). For the anatomical experiments where different constructs were compared, we mixed the components with volume ratios adjusted so that the titer of the combined injectate was 1.10^11^vg/ml for all conditions. Mixes of tTA and TRE-GCaMP6s viruses were used with a 1:1 ratio.

The appropriate injectate mixes were backfilled into quartz capillary pipettes designed for targeting IO (Guo et al., 2021). The pipette was slowly inserted in brain tissue (~ 0.2 mm/s) to the target locations. In every animal, a total of 4 injections was made, 2 on each side. The first targeted the principal and dorsolateral IO subnuclei (Bregma-origin coordinates: anterio-posterior (AP):-6.2 mm, medio-lateral (ML): 0.42 mm, dorso-ventral (DV):-6.7 mm) and the second one was purposefully made slightly off-target (AP: -6.2 mm, ML: 0.42 mm, DV: -6.6 mm). At these locations, 200 nl of the viral solution was discharged from the pipette at 40 nl/min. In total, each animal received 800 nl of viral injectate at these four locations. After withdrawing the injection pipette, the skin was cleaned and carefully closed with cyanoacrylate glue. The mouse was subcutaneously administered 5 mg/kg of the analgesic drug Rimadyl (Zoetis, New Jersey, US) together with 0.2 ml of saline (NaCl 0.93%) to prevent dehydration. Notably, we did not administer D-mannitol as is often done to induce systemic hyperosmolarity and enhance viral spread (Burger et al., 2005) as we did not wish to cause additional physiological stresses to the animals.

### 2.4. Anatomical Study of Transgene Expression in IOn: Virus Preparation and Injection

For anatomical quantification of transfection results, animals were intracardially perfused 30 days after injection using at least 50 ml of 4% paraformaldehyde in phosphate-buffered saline (PBS; pH 7.4). After fixation, the brain was extracted and immersed for 6 h in the same PFA solution before washing in PBS. Coronal brainstem and sagittal cerebellar sections (80 μm thick) were cut with a vibratome (5100MZ-plus; Campden Instruments, Loughborough, UK) equipped with ceramic blades (38 x 7 x 0.5 mm ceramic blades, model 7550-1-C, Campden Instruments, Loughborough, UK) and mounted on objective glass with Vectashield (H-1200, VectorLabs, CA) mounting medium and # 1.5 coverslip glass (Harvard Apparatus, MA).

### 2.5. Immunohistochemistry

For histological labeling of astrocytes, 100 μm thick coronal brainstem slices were prepared from perfusion-fixed P40 C57ML/6J male mice (CLEA Japan, Shizuoka, Japan). The slices were permeabilized by incubation in GSA-BSA-T - PBS solution with 10% BSA (Bovine Serum Albumin, Sigma-Aldrich, Germany), 1% GSA (Goat serum, normal donor herd, Sigma-Aldrich, Germany), and 0.1% Triton (Sigma-Aldrich, Germany) for 2 h on a shaker (40 rpm) at room temperature (25°). For primary antibody application, 200 μl of a mix containing Anti-NeuN (1/200, Polyclonal Guinea pig antiserum 266-004, Synaptic Systems, Germany), Anti-MAP2 (1/200, Polyclonal Chicken antibody, ab5392, AbCam, Japan) and Anti-AlphaTubulin (1/100, Monoclonal mouse antibody, CP06, Sigma-Aldrich, Germany) diluted in BSA-GSA-T was deposed on top of the slices in individual wells and slices were incubated for 15 h. After washing the slices 4 times with BSA-GSA-T, the slices were incubated with 3 secondary antibodies for targeting the 3 primary antibodies coupled to a different fluorescent reporters (Anti-mouse-Alexa488, Anti-guinea pig-Alexa555, Anti-chicken-Alexa591, ThermoFisher Scientific, Minato, JP) for 4h, rinsed again 4 times in PBS and prepared for microscopy.

### 2.6. Confocal Scanning of Anatomical Samples

**Figure 1:**
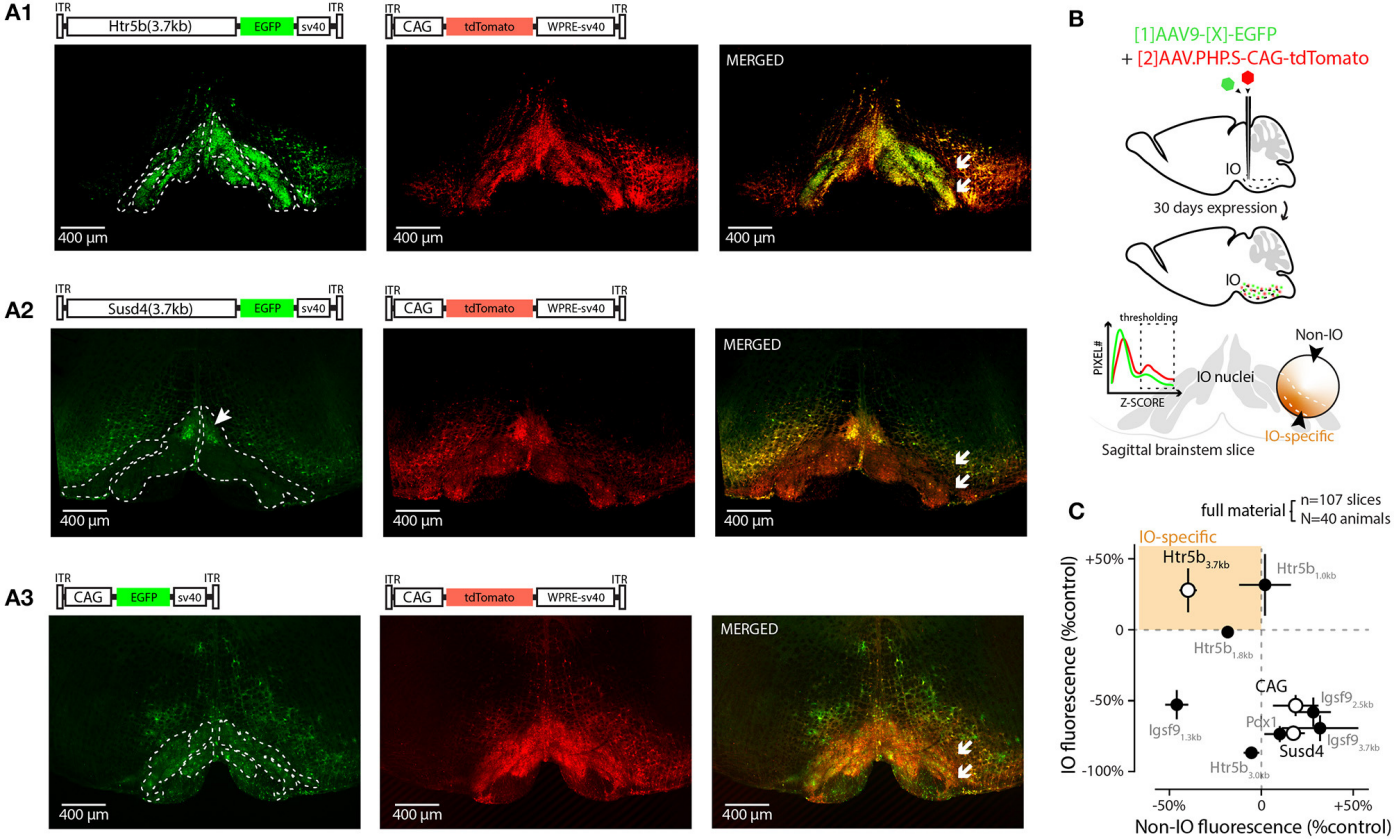
Screening 10 viral constructs for efficient and selective transgene expression in the inferior olive. **(A)** Confocal images showing EGFP (left) fluorescence driven with three example promoters (Htr5b(3.7), Susd4(3.7), and CAG in **(A1,A2,A3)**, respectively). Middle shows the tdTomato fluorescence driven with the reference construct (AAV.PHP.S-CAG) that was used to normalize the intensity measurements in each sample; the merged fluorescence images are shown on right. White arrows in merged images indicate the sites of injection on one side of IO, showing that the injections included a purposeful “mistargeting” for more fair assessment of labeling specificity. The plasmid used for each construct is schematically depicted above the confocal images. **(B)** Schematic describing the definition of IO-specific and non-specific measurements. **(C)** Summary of all the 10 constructs' normalized expression strengths inside and outside of the IO (vertical and horizontal axes, respectively). Only constructs based on clones of the serotonin receptor 5b (Htr5b) show similar or better performance in the IO than the reference construct. The results from the three example constructs shown in **(A)** are indicated by white markers. The error bars denote population SEMs.

**Figure 2:**
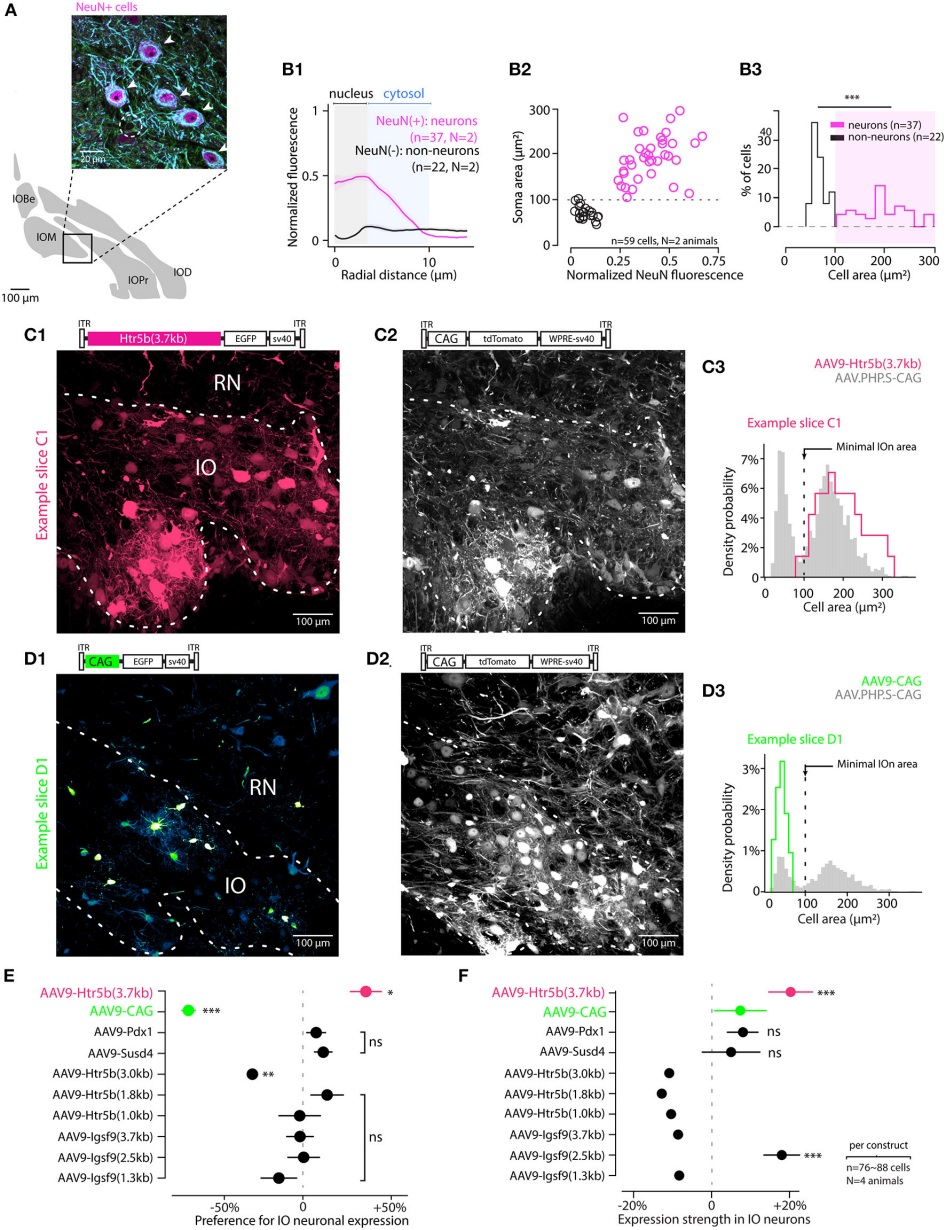
Screening viral constructs for specific expression in IO neurons over astrocytes. **(A)** Identifying IO neurons and astrocytes in triple-antibody-labeled slices. The neurons are identified with NeuN and MAP2 immunoreactivity (purple and blue channels; somata indicated with arrowheads in the enlarged section of confocal image). Astrocytes are only labeled with α-Tubulin (green; cell soma indicated with a dashed line). **(B)** Using NeuN staining as a criterion for defining cells as neurons as opposed to non-neurons based on soma area. **(B1)** Averaged radial NeuN staining intensity plots for cells classified as neurons or non-neurons (normalized somatic NeuN >or < than 20%, respectively). The shaded regions highlight radial regions corresponding to the neuron nuclei (gray) and cytosol (blue). **(B2)** Scatter plot showing the relationship between cell soma area and the normalized NeuN staining intensity for neurons (purple) and astrocytes (black). Dashed line indicates the threshold value of 100 μm^2^ used to classify cells as neurons or non-neurons. **(B3)** Histogram of the soma areas classified as either neurons (purple) or non-neurons (black). **(C,D)** Example confocal images and cell type identification for two of the 10 constructs tested. Top shows the GFP image channels, obtained from IO injected with AAV9-Htr5b(3.7) and AAV9-CAG (**C** and **D**, respectively), and middle show tdTomato channel confocal scan, reporting for the reference construct (AAV-PHP.S-CAG) expression. Both images are maximal *z*-projections of a 30 μm thick stack volume. Right **(C3,D3)** show histograms for labeled soma sizes obtained for the two example constructs (red and green histograms), as well as those for the control constructs (gray histograms). Dashed vertical line denotes the 100 μm^2^ soma area threshold used to classify cells as either neurons or astrocytes. Note that the color scales in each confocal image are adjusted for visual clarity, and quantitative assessments are done based on the numerical comparisons as shown in other. **(E)** Summary of the cell-type preference of the 10 constructs, where expression in neurons is normalized to expression in non-neurons in the same slices. The ratio was calculated per-animal on ~ 80 astrocytes and 80 neurons per animal. Each dot is the average of neuron-to-astrocyte ratio per animal and the value is an indicator for neuronal expression performance in IO. The statistics compare cell-type-preference of the test virus to the reference virus (AAV.PHP.S-CAG-tdTomato) in material where both vectors are co-expressing tdTomato and EGFP transgenes. The only construct with clear preference for IO neurons was the AAV9-Htr5b(3.7) (Welch t-test, t = 2.93, p = 0.026). **(F)** Summary of the normalized expression strength in neuronal cells among the 10 constructs (76 to 88 neurons were selected in each construct using the 100 μm^2^ surface threshold). Neuronal expression of test transgene was normalized to the expression of reference transgene (AAV.PHP.S-CAG-tdTomato) for each neuron, then the population was averaged. Statistics compare the normalized expression of high-expression test vectors to the normalized expression AAV-CAG-EGFP transgene. The constructs to the right of the dashed vertical line are expressed stronger in neurons than the reference construct. The difference of expression between test transgene and control transgene in neurons co-expressing both proteins is stronger in AAV9-Htr5b(3.7) compared to AAV9-CAG (Welch *t*-test, t = -6.09, p < 0.001). EGFP is significantly more expressed in neurons co-infected with AAV9-Htr5b(3.7) and the reference virus. In **(E,F)**, horizontal lines denote population SEM ns, p > 0.05.

**Figure 3:**
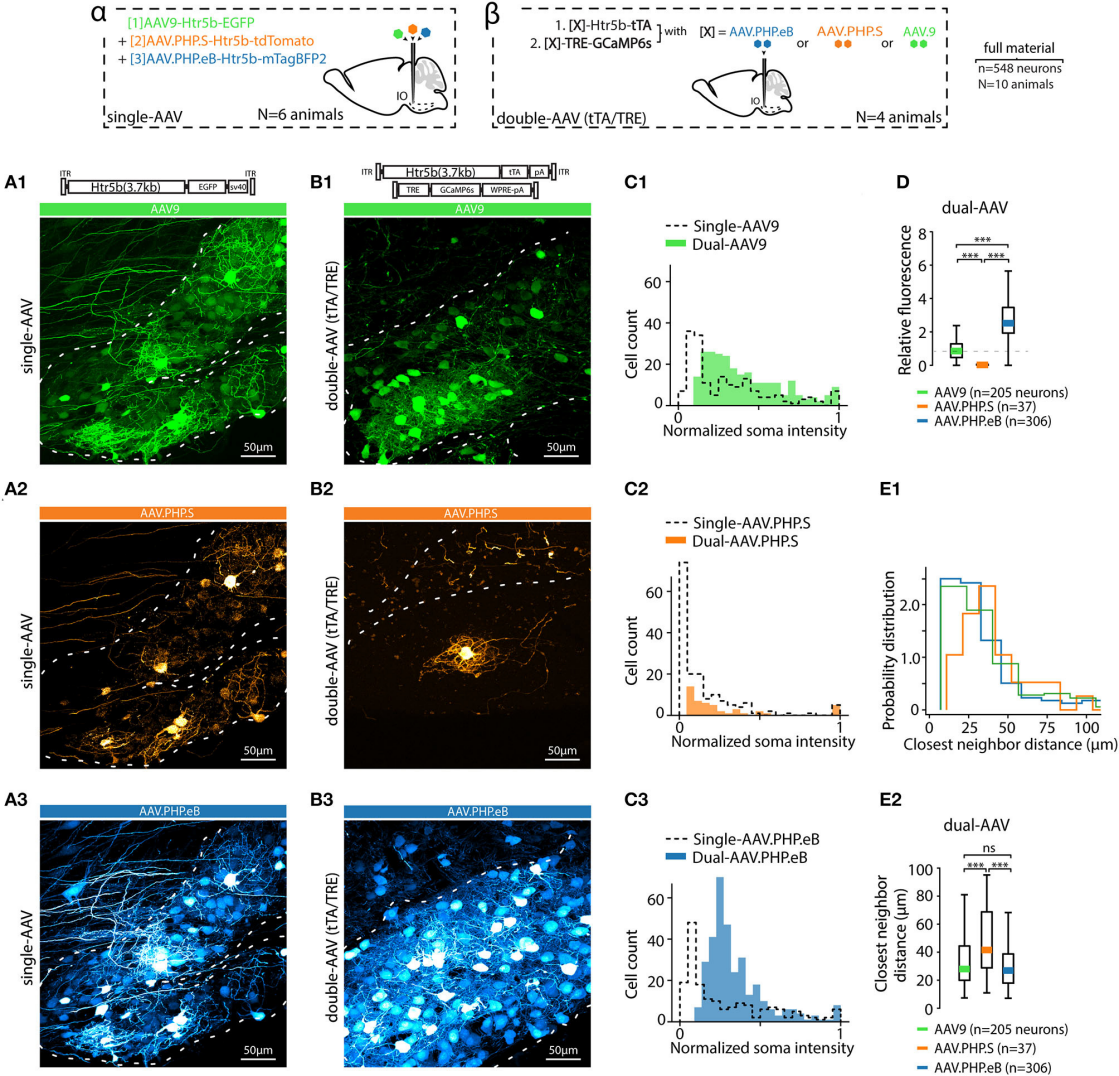
AAV capsid type influences transgene expression with both single and dual-AAV approach. **(A)** Example confocal images of the IO acquired from an animal where three different fluorophores (EGFP, tdTomato and mTagBFP2) were expressed under the control of Htr5b(3.7) promoter but with different AAV capsids (AAV9, AAV.PHP.S, and AAV.PHP.eB in **(A1,A2,A3)**, respectively) after 4 weeks of transfection. Dashed lines indicate the contours of the IO. A schematic labeled *alpha* on top of the figure depicts the design of the experiment. **(B)** Example confocal images acquired from the IO of three different animals, in each of which a dual-AAV mixture utilizing different viral capsids (AAV9, AAV.PHP.S, and AAV.PHP.eB in **(B1,B2,B3)**, respectively) were used to deliver the sequences for expressing Htr5b(3.7)-tTA/TRE-controlled GFP. A schematic labeled *beta* on top of the figure depicts the experiment design. Note that the color scales in each example confocal image are adjusted for visual clarity, and quantitative assessments are done based on the numerical comparisons as shown in other. **(C)** Distributions of single-soma fluorescence intensities from n = 548 cells from 20 slices in 10 animals, normalized to maximal values for the three different viral capsids. Dashed lines denote data obtained with single virus experiments, solid color bars are from dual-AAV experiments. **(D)** Comparison of the mean fluorescence intensities between samples obtained with the three capsids. Mean values, indicated by colored bars, are normalized to the mean value of AAV9 intensities (dashed line). **(E1,E2)** Comparison of the shortest distances between neighboring labeled somata with the three capsids. (1-way ANOVA, [AAV9 vs. AAV.PHP.S] f = 14.5, p < 0.001; [AAV9 vs. AAV.PHP.eB] f = 0.3, p = 0.86; [AAV.PHP.S vs. AAV.PHP.eB] f = 13.9, p < 0.001).

**Figure 4:**
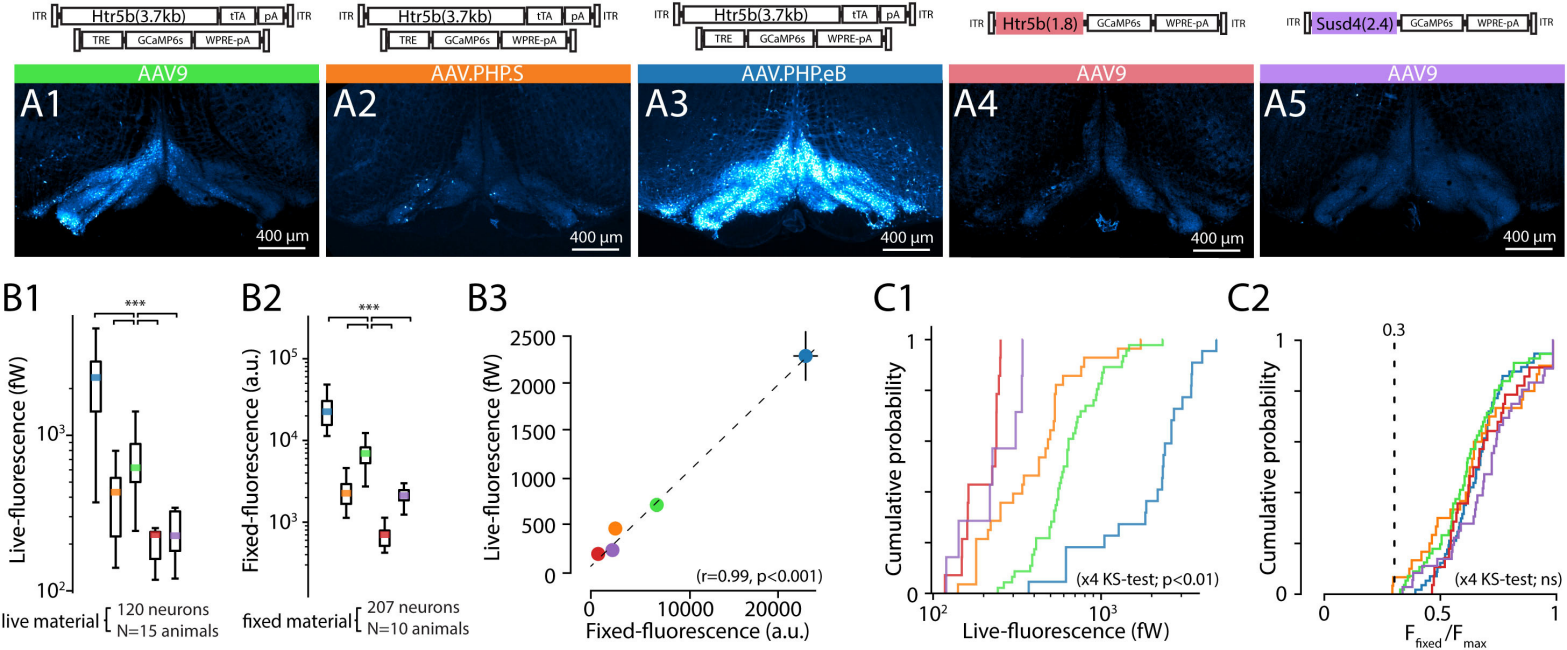
Comparison of tissue-fixed and live-imaging GCaMP6s fluorescence intensities *in-vitro*. **(A1–A5)** Example confocal images (5x) demonstrating wide-area GCaMP6s fluorescence obtained with 5 different constructs (as indicated above each) after 2 weeks of expression. Images are acquired from 300 μm brainstem slices, immersion-fixed after live *in-vitro* imaging experiments. **(B)** Comparison of the constructs' baseline (F_0_) whole-field live fluorescence measurements for individual neurons **(B1)** and confocal imaging **(B2)** before **(B1)** and after **(B2)** immersion-fixing. **(B3)** shows the high correlation between the live imaging and confocal imaging results. Colored dots are average values for each construct, where 10 to 20 neurons per slice for the live, and fixed condition in the same slices. (n = 3 slices per animal, min. 2 animals per construct). **(C)** Distribution of raw live-imaging fluorescence intensities varies between constructs **(C1)**, but the relative ranges are nearly identical when normalized to maximal intensity in each slice **(C2)**. Dashed line in **(C2)** indicates relative intensity of 0.3. For **(B,C)**, data from different constructs are labeled with colors as in **(A)**.

**Figure 5:**
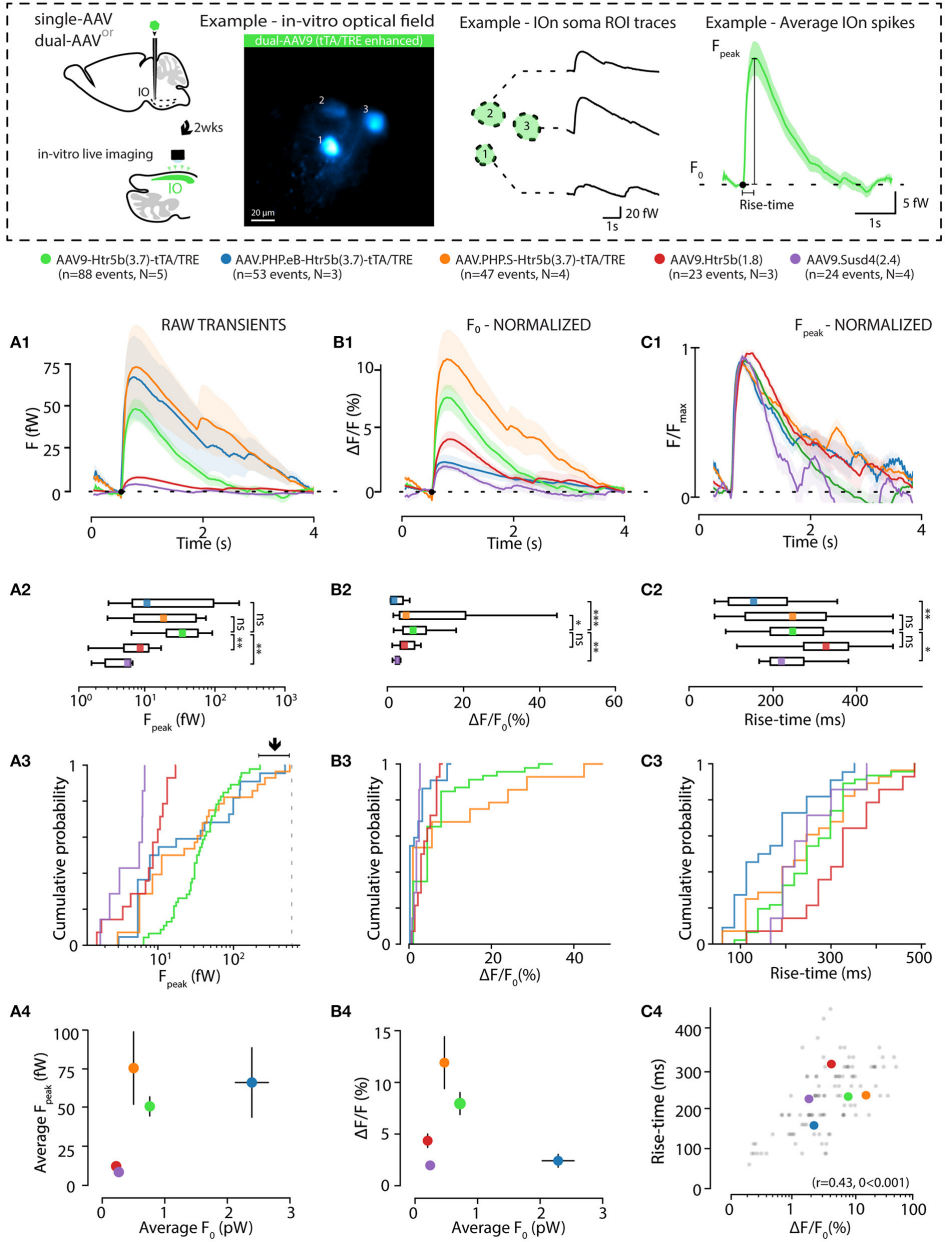
Effects of GCaMP6s expression level on the action-potential-related fluorescence signal. **(A,B)** Comparison between calcium events in units of power (fW) obtained with the 5 different constructs as indicated by the color labels, 2 weeks after injection. The schematic examples on top of the figure (in a dashed-line box; note that the image brightness of the standard deviation time series projection is adjusted for viewing and should not be considered as representative of the dynamic range during live imaging) describe extraction of the calcium fluorescence event parameters in acute IO slices. **(A1)** Averaged event waveforms aligned on initiation point (black dot). The shaded areas denote ± SEM. **(A2)** Average calcium event peaks; **(A3)** their cumulative distributions, with arrow and horizontal line segment highlighting the convergence of maximal values of eCa peaks between high-expressing constructs; **(A4)** relation between average event peaks and baseline fluorescence in each cell. **(B)** Same data as in **(A)** but normalized to F_0_ for a baseline-normalized DFF values, similarly represented in boxplot **(B2)**, distribution plot **(B3)** and construct-averages represented against their respective F_0_s **(B4)**. **(C)** Same data as in **(B)** but peak-amplitude-normalized to compare event kinetics. **(C1)** averaged event waveforms; **(C2)** comparison of average event rise times; **(C3)** cumulative distributions of event rise times; **(C4)** individual event rise times and baseline-normalized event amplitudes show linear relation. The colored dots in **(C4)** represent averages for each construct data, gray dots are individual events. Colored bars in **(A2,B2,C2)** denote average values. Horizontal and vertical lines in **(A4,B4)** denote ± SEM values in each dimension. Statistics for eCa F-peak on **(A2)** (1-way ANOVA, [AAV9-Htr5b(3.7)-tTA/TRE vs. AAV.PHP.eB-Htr5b(3.7)-tTA/TRE] f = 1.56, p = 0.1; [AAV9-Htr5b(3.7)-tTA/TRE vs. AAV.PHP.S-Htr5b(3.7)-tTA/TRE] f = 1.9, p = 0.17).

Confocal image stacks were acquired from sections labeled with viral or immunohistological methods with a Zeiss LSM 880 confocal system (Zeiss, Germany). For low-magnification imaging (mesoscopic; Figures 1, **4**), 5x objective (Plan-Apochromat 5x M27; NA 0.16; Zeiss, Germany) and 5-10 μm z-step were used. For high-magnification images (Figures 2, 3, **12**), 40x objective (Objective “Plan-Apochromat” 40x Oil DIC M27; NA 1.4; using Zeiss Immersoil oil; Zeiss, Germany) with (1 μm z-steps, at least 30 μm thickness of tissue section) was used. For multi-channel images, acquired in line-scan mode, the following excitation/emission wavelengths were used: EGFP, Argon 488 nm / 490–535 nm; tdTomato, DPSS 561 nm / 470–655 nm; mTagBFP2, UV-DIODE 405 nm / 420–480 nm. We took special care to match acquisition parameters for different trials of the same experiment set.

### 2.7. Preparation of Acute IO Slices for *in-vitro* Calcium Imaging and Patch-Clamp Experiments

For *in-vitro* calcium imaging, animals that had been injected with the viral constructs to express GCaMP6s were used after 2 weeks of transfection time. The acute IO slice preparation has been previously described in detail. In brief, the animals were anesthetized with lethal dose of isoflurane and decapitated. Brainstem is extracted in warm standard physiological solution (SPS; composition: 126 mmol NaCl, 10 mmol glucose and 26 mmol NaHCO_3_, 3.4 ml KCl, 1.2 ml KH2PO_4_, then 1.3 ml MgSO_4_ (1 M), 2 mL CaCl_2_ (1 M); pH to 7.2–7.3; 34°C). 300 um thick coronal brainstem slices were cut with a vibrating microtome (7,000 smz-2, Campden Instruments, UK) equipped with ceramic blades (model 7550-1-C, Campden Instruments, UK) at low slicing speed (0.01 mm/s). After at least 1 h recovery period, slices were transferred to a submerged-type recording chamber, continuously perfused with warm (34°C) SPS. Slices were viewed with an upright microscope (BXW51, Olympus, JP) with 5x (NA:0.1, MPLN5X, Olympus, Japan) and 60x (NA:1.0, LUMPLFLN60XW, Olympus, Japan) objectives.

To excite GCaMP6s fluorescence, we used whole-field illumination with a 488 nm LED light (pE-300 ultra, CoolLED, UK) and filtered with an EGFP filter cube set (457–538 nm, U-FF, Olympus, Japan). 10-s fluorescence image sequences were acquired at 30 frames per second (fps), 256x256 resolution (1.22 um pixel size for 60x objectives) with a CMOS-based MiCAM03 imaging system (BrainVision, Japan). Care was taken to suppress mechanical movement of the slice or perfusion liquid during recordings. For each tested construct, several LED illumination intensities were tested (10, 40, 80, 100 mW illuminations) to avoid sensor saturation and to keep the light arriving to camera sensors below 60% of full-well capacity.

A second camera (sCMOS Zyla4.2, Andor, UK) was used to target neurons for simultaneous patch-clamp and calcium imaging recordings. Neurons were included or excluded from the study based on their quality score [see Dorgans et al. (2020)]. Cells were patched with filamented borosilicate pipettes (unpolished capillaries with filament and with outer and inner diameters of 1.5 and 0.86 mm, respectively; 10 MOhm; BF-150-86-10, Sutter Instrument, USA) filled with intracellular solution containing 4 mM NaCl, 0.001 mM CaCl_2_, 140 mM K-gluconate, 0.01 mM EGTA, 4 mM Mg-ATP, 10 mM HEPES, osmolarity adjusted to 310 mOsm with K-gluconate and pH to 7.2 with KOH. IO neuron activity was recorded in current-clamp mode at 50 kHz sampling rate, amplified, digitized and low-pass-filtered at 5 kHz with patch-clamp amplifier (Double IPA Integrated Patch Clamp Amplifiers with Data Acquisition System, Sutter Instrument, USA). In a few cells, 200 pA current pulses were used to evoke spikes. For the double patch-imaging recordings used for characterizing the spike-waveform properties of corresponding calcium events, we only selected neurons with spontaneous spiking activity seen over a low-fluorescent baseline level.

Following the *in-vitro* experiments, slices were fixed by immersion in 4% PFA for exactly 1 hour. After a series of washouts (at least 3 x 10 min in PBS), slices were mounted with ProLong Glass antifade medium (ThermoFisher Scientific, Minato, Japan) for confocal imaging. These slices were used to compare paraformaldehyde-fixed levels of GCaMP6s fluorescence and live GCaMP6s (average background fluorescence: see Calcium imaging analysis method section for details).

### 2.8. *In-vivo* Imaging of IO Calcium Activity

For imaging IO calcium activity in a living mouse, we used our recently-described surgical procedure that exposes the ventral side of the brainstem (Guo et al., 2021), allowing optical access to the inferior olive with a GRIN-lens-coupled miniature microscope (nVoke2, Inscopix, CA; excitation light: 455 ± 8 nm; emission bandwidth: 515 ± 25 nm). Briefly, 2 to 3 weeks after virus injection, the mouse was anesthetized with isoflurane, attached to a stereotactic frame with ventral side up, and the tissues overlaying the IO were surgically removed. Next, the miniscope with GRIN lens (width: 1 mm, NA:0.5 in water) was positioned on the surface of the IO without damaging the *dura mater*. For the cerebellar cortical recordings, the mouse was attached similarly to the stereotactic frame but with dorsal side up. A craniotomy (less than 2 mm wide) was opened over the lobules V–VII of cerebellar cortex with a hand-held dental drill. The miniscope and GRIN lens were gently placed on the surface of the cerebellum to avoid damage to climbing fiber branches by osmotic changes or mechanical damage. For both approaches, when fluorescent cell bodies or axonal branches were in focus, 20–30 fps image series (2.96 pixel per μm) were acquired using the miniscope software (Inscopix data acquisition software, nVoke acquisition system, Inscopix, CA). The animal was kept under deep anesthesia throughout the experiment. After experiment was completed, a lethal dose of isoflurane was administered and the brain was prepared for anatomical study by transcardiac perfusion as described above.

### 2.9. Identifying Astrocytes and Neurons With IHC Labeled IO

To compare sizes of IO neurons and IO non-neurons, contours of individual cells (regions of interest, ROIs) were manually traced from maximal projection images 40x z-stacks of merged Anti-MAP2, Anti-alpha-Tubulin and anti-NeuN channels. Neurons were identified by their clear somatic boundaries and NeuN staining, while non-neuronal cells had uneven shapes and appeared dark in alpha-Tubulin channel. Radial intensity profile plots centered on soma center were acquired in regions where the three immunohistological labels were uniformly labeling the 30- μm-thick z-stack, using *Radial Profile Angle* plugin in **FIJI** software (ImageJ, U. S. National Institutes of Health, USA). Values were normalized to the maximal intensity peak of each plot profile from each channel. A cell was classified as a neuron if the normalized NeuN staining exceeded 20% within 2 μm radius. All other cells were classified as “astrocytes,” even though it is possible other non-neuronal cells would be present. Cell areas were calculated from the manually-drawn ROIs.

### 2.10. Quantifying Structural Specificity of the Constructs

To provide comparative data for the constructs' preference for IO over the surrounding brain regions, we used maximal intensity projections of 5x confocal stacks (40 μm depth) obtained with EGFP and tdTomato as described above. First, the IO structural contours were manually drawn in FIJI, and intensity histograms for the two channels were obtained for regions both inside and outside of IO. Next, histograms were thresholded (Z = 3) and relative expression levels inside and outside IO were calculated as ratios of spatial extents, as well as intensities of the labeling.

### 2.11. Quantifying Transgene Expression in IO Neurons and Astrocytes

The amount of light emitted by a cell expressing a fluorescent reporter is dependent on its concentration within a volume of brain tissue and sensitive to experimental bias. For example, variation in target location, as well as the diffusion of viral particles within the tortuous brain tissue create local variations of AAV concentrations *in-situ* and may affect the amount of transgene expression. We aimed for high reproducibility and assessment of IO neuron targeting by comparing fluorescence intensity obtained with each “test-construct” expressing EGFP with a “reference construct” expressing tdTomato.

For single-soma expression measurements in fixed samples, average intensities of hand-drawn ROIs were acquired for the test-transgene (EGFP), as well as those for the control-transgene (tdTomato). Neurons separated from astrocytes based on the 100 μm^2^ soma area threshold. We used maximal intensity projection image from z-stack acquisitions of lateral (IOPr, IOD) and medial IO nuclei (IOM, IOBe). All z-stacks were at least 20–30 μm so that they included at least one full neuron soma. The contours of individual somata were manually drawn observing the fluorescence intensity in combined EGFP and tdTomato channels, and confirmed with a normalized, equalized and subtracted channels. Average intensity value was obtained for each ROI in each channel. Finally, EGFP intensity was divided by tdTomato intensity to provide a normalized expression value. For estimating expression level of GCaMP6s in post-fixed acute slices ‘Fzero-fixed', average ROI intensity values from confocal images scanned from immersion-fixed slices from *in-vitro* experiments were used. For calculating the nearest neighbor distance between observed neurons, the shortest Euclidean distances between the center coordinates of neuron ROIs were used.

### 2.12. Calcium Imaging Analysis

To extract somatic calcium signals from image time series acquired with the MiCam03 system, soma ROIs were manually drawn in Fiji based on standard deviation and maximal projections to improve accuracy and to only include cells that presented calcium fluctuations. ROIs with areas smaller than 100 μm^2^ (non-neuronal cell soma) were excluded. The intensities of all pixels within a ROI were averaged for a single-frame raw value. To allow comparison between experiments, the raw digital intensity values were converted to light power (fW) using the well-depth values (600.000 e^-^ full-well, 130 e- dark noise) provided by camera specification sheet (https://www.scimedia.com/fis/neuro/mc0503spec) and eV constant (1.6.10^-19^J) as follows:


$$F_{\text{live}}(W)(J.s^{-1})\;=\;e^{-}{\;}_{\text{pixel}}.1eV(J).sf\;(s^{-1})$$

This conversion to fW is informative of the measured light power and can be used to compare recordings with different frame rates, even though it doesn't represent the real amount of light emitted by the recorded neurons.

Calcium events (eCas) were detected from the somatic fluorescence recordings based on maximal instantaneous slope increase (with a Z-threshold = 4) and minimal 500 ms peak-to-peak separation. Notably, IO neurons' spontaneous firing rate rarely exceeds 0.2 Hz *in-vitro*, and thus the slow decay kinetics of the GCaMP6s are not a source of concern. For waveform analysis, calcium traces were aligned on the spike onset, baseline values ‘F_zero_-live' were calculated as the minimal value observed in the time window 500ms before spike onset. eCa rise times (RT) were calculated as the time between spike onset and transient peak occurrence. eCa peak amplitude was taken as the maximal fluorescence peak intensity of the extracted signal. Double-events and other false positives were manually discarded. STO-PSD (power spectral density) calculations were made on the band-passed (3–12 Hz) signals using Blackman-windowed Welsch method, taking care of subtracting outflanked decaying slopes of IO spikes peaks in the 0–3 Hz bands to avoid false-positives. All extracted power spectra were checked manually for other artifacts before further analysis.

### 2.13. Analysis of Membrane Voltage Recordings

Continuous membrane voltage (V_*m*_) recordings in current-clamp mode were sampled at 50 kHz and recorded with SutterPatch Software (v2, Sutter Instrument, USA) running on IgorPro Software (v7, WaveMetrics, Oregon, US), that was used to trigger shorter-duration acquisition of calcium images with a fixed and recorded delay, accounting for mechanical shutter delay. The V_*m*_ traces were segmented and aligned based on detected eCas and IO spike width was defined as the duration of the calcium shoulder when the spike waveform initial depolarization slope reaches a +10 mV threshold. STO power analysis was done using Blackman-windowed Welch method as for the imaging data above. PCA were computed from the raw, aligned V_*m*_ spike waveform, k-means clustering was done on the first 2 PCA components and the optimal number of clusters was selected with elbow-method (Liu and Deng, 2021).

### 2.14. Data Processing and Statistics

All statistical analysis was done with custom-written scripts in Python language (WinPython Spyder3.2.2, Python 3.6.7), utilizing the following plugins: *numpy 1.19.1* (basic operations), PIL plugin (python image library), *scipy 1.5.2* (correlations, cross-correlations, resampling, statistics) and *pandas 0.23.4* (data management, time-lagged cross correlations), *scikit-learn 0.23.2* (PCA). The code used to create the analysis for this work is available online in repository https://github.com/Dorgans/IO_specific_promoters. Additional image processing was done in FIJI (ImageJ, U. S. National Institutes of Health, USA, Schindelin et al., 2012). Voltage traces were processed with Neo plugin (v0.11, https://neo.readthedocs.io/en/latest/), Python language, Garcia et al. (2014).

All data are given as mean ± SEM. Normality of distribution was checked with the Pearson-d'Agostino omnibus test of normality combining skew and kurtosis. Variance between individual observations of group data were tested with Levene test for variance equality between two groups. In case of equal variances, group data were tested with Student t-test or ANOVA as indicated. In case of unequal variance, Welch t-test was used. Distributions were compared with the Kolmogorov–Smirnov KS-test. Statistical significance was defined at 4 levels (1: p < 0.05, 2: p < 0.01, 3: p < 0.001 or N.S: p >0.05).

## 3. Results

### 3.1. Selection of Candidate Viral Constructs for Efficient Transgene Expression Targeting in the Mouse Inferior Olive

To create efficient viral constructs for transfecting IO neurons, we used knowledge from past literature describing IO-specific gene expression of the following 4 promoters (see Table 1 for sequence information):

1. Igsf9 (Immunoglobulin Superfamily Type 9), expressed in the developing central nervous system and related to the expression control of cell-adhesion molecules (Hansen and Walmod, 2013). Igsf9 has a strong expression in human and mouse IO nuclei (Doudney et al., 2002; Pätz et al., 2018). We cloned 3 promoter versions of Igsf9 gene (1.3, 2.5, 3.7 kb).
2. 5-hydroxytryptamine (serotonin) receptor 5B, that is weakly expressed in the mouse CNS but presents a strong expression in IO (Tanaka et al., 2012; Good et al., 2017). We cloned 4 promoter versions of Htr5b gene (1.0, 1.8, 3.0, 3.7 kb).
3. Pdx1 (Pancreatic and Duodenal Homeobox 1; Song et al., 2010; Vrieler et al., 2019), expressed during development for organ morphogenesis and maintains its expression in the adult mouse in some populations of cells (including IO neurons).
4. Susd4 (Sushi Domain Containing 4; González-Calvo et al., 2021) gene promoter is driving expression of adhesion molecules in the axons of IO neurons.
5. The synthetic CAG-promoter, is commonly used for driving gene expression by viral approaches we used it as a control promoter (Hitoshi et al., 1991).

As a first step in evaluating the potential of these promoters and to provide quality control for our in-house-made vectors, viral vectors driving expression of enhanced green fluorescent protein (EGFP) under each of the 9 constructs above were injected into and near the IO of adult mice at 1:1 ratio with a commercial, general-purpose expression vector driving tdTomato reporter under AAV-PHP.S-CAG-tdTomato (Addgene#59462; Chan et al., 2017); “reference virus.” This allowed quantification of the different constructs' expression within the ventral brainstem across animals (N = 4 animals for each of the 9 constructs) by normalizing EGFP fluorescence intensities to tdTomato. Figure 1 shows low-magnification confocal images **(A1–A3)** of the EGFP and tdTomato channels for coronal brainstem sections (5x magnification) from three different experiments for the mesoscale expression quantification (see schematic diagrams in Figure 1B). The extent of EGFP fluorescence varied among the different constructs in terms of preference toward the IO or the neighboring structures (Figures 1A1–A3, left), whereas the AAV.PHP.S-CAG virus always labeled a rather continuous area of the ventral brainstem around the injection site, with no evident bias between the IO and the surrounding neurons of the reticular nucleus or raphe nuclei (Figures 1A1–A3, middle). To provide quantitative comparison of the individual constructs' potential for targeting the IO, the GFP expression was compared to the AAV-PHP.S-CAG-driven expression of tdTomato (see merged fluorescence channels in Figure 1A, right). Normalizing the extent of GFP expression to the area labeled by tdTomato, we found that while multiple constructs could label structures outside of the IO stronger than the AAV-PHP.S-CAG (Figure 1C, datapoints to the right of the vertical dashed line), only those built on clones of the Htr5b-promoter performed equally well or better within the IO than the AAV-PHP.S-CAG (Figure 1C, datapoints above the horizontal dashed line; see Table 2 for individual values). Most of the constructs, including Igsf9, Pdx1 and Susd4 (that have previously been shown to drive IO-specific transgene expression when used in cre-transgenic animals) drove stronger overall expression in the regions surrounding the IO (Figure 1C, datapoints to the right of the vertical dashed line). Notably, GFP-expressing IO cells were seen densely labeled in limited region of the dorsomedial IO (2 out of 4 mice, subnucleus IO-Be, see arrow in Figure 1A2) with the Susd4-promoter (González-Calvo et al., 2021), but the expression was seen only sparsely within the other IO subnuclei.

**Table 2:** Anatomical characterization results for transgene expression in inferior-olive with custom AAV vectors.

**Construction**	**Normalized IOn expression strength (%)**	**Cell-type specificity (% IOn/non-IOn)**
AAV9-Igsf9(1.3)-EGFP	-8.77 +/- 0.085 (n = 80)%	-15.3 +/- 11.3%
AAV9-Isgf9(2.5)-EGFP	+17.54 +/- 4.57 (n = 80)%	+0.47 +/-10%
AAV9-Isgf9(3.7)-EGFP	-9 +/- 0.8 (n = 80) %	-1.85 +/- 8.4%
AAV9-Htr5b(1.0)-EGFP	-10.85 +/-0.28 (n = 88)%	-2.05 +/- 13.16%
AAV9-Htr5b(1.8)-EGFP	-13.25 +/- 0.16 (n = 79)%	+15.4 +/- 10.3%
AAV9-Htr5b(3.0)-EGFP	-11.3 +/- 0.85 (n = 81)%	-32.13 +/- 1.84%
AAV9-5HTr2b(3.7)-EGFP	+19.8 +/- 5.6 (n = 79)%	+40 +/- 9.8%
AAV9-Pdx1-EGFP	7.59 +/- 3.92 (n = 77)%	+8.42 +/- 5.95%
AAV9-Susd4-EGFP	4.5 +/-7.39 (n = 82) %	+13 +/- 05.6%
AAV9-CAG-EGFP	6.5 +/-6.63 (n = 76)%	*-72.8* +/- 3.67%

**Table 3:** *In-vitro* properties of GCaMP6s signals found in inferior olive.

**Construction**	**Average baseline fluorescence (Fzero, fW)**	**Average spike transient amplitude (Fpeak, fW)**	**STO amplitude RMS (fW^2^/Hz)**
AAV.PHP.eB-Htr5b(3.7)-tTA/TRE-GCaMP6s	2711 +/- 309 (n = 29) fW	66.6 +/- 19 fW	3.19 +/- 0.6 (n = 29) fW^2^/Hz
AAV9-Htr5b(3.7)-tTA/TRE-GCaMP6s	432.7 +/- 65.1 (n = 29) fW	74.1 +/-23.7 fW	0.4 +/- 0.2 (n = 15) fW^2^/Hz
AAV.PHP.S-Htr5b(3.7)-tTA/TRE-GCaMP6s	735 +/- 58 (n = 50) fW	45.5 +/- 5.5 fW	0.02 +/- 0.1 (n = 28) fW^2^/Hz
AAV9-Htr5b(1.8)-GCaMP6s	201.5 +/-13 (n = 14) fW	8.9+/- 1.13 fW	0.0018 +/- 0.0002 (n = 2) fW^2^/Hz
AAV9-Susd4(2.4)-GCaMP6s	255.4 +/- 25 (n = 12) fW	5.5 +/- 0.8 fW	0.053 +/- 0.03 (n = 12) fW^2^/Hz

Thus, in the group of 10 promoters tested (Figure 1C) with an AAV9 capsid, the longest clone of the Htr5b promoter drove expression in most IO-selective manner (i.e., strongest expression within the IO and lowest expression in the surrounding structures, compared combined reference virus patterns; 1-way ANOVA, f = 19.75, p < 0.001, n = 10, N = 3). Intriguingly, we saw a significantly lower expression in the IO when using the CAG-promoter in an AAV9 capsid when comparing with the PHP.S (1-way ANOVA, f = 7.88, p < 0.05, n = 8 slices, N = 3), suggesting that combinations between viral capsid and promoter influence the targeting of gene expression to the IO neurons (Powell et al., 2020).

### 3.2. Optimizing Viral Expression for Neurons Over Astrocytes

Next, we investigated the transgene expression driven by the constructs in neurons compared to non-neuronal cells. To determine morphological features that distinguish neurons from astrocytes (“non-neurons,”) we performed triple immunohistochemistry on perfusion-fixed IO slices (Figure 2A). Using antibodies that are specific (Anti-MAP2, Anti-NeuN) and non-specific (Anti-*alpha*-Tubulin) for neurons. In such samples, astrocytes could be clearly distinguished from neurons (arrowheads in Figure 2A; an astrocytic soma is indicated with dashed line) based on the absence of NeuN immunoreactivity in the cell nucleus (Dorgans et al., 2020). We then measured the intensity of NeuN-immunofluorescence within the radius of IO somata (Figure 2B1) and plotted it against the soma areas (Figures 2B1,B2). As the 5th and 95th percentiles of soma area distributions for NeuN-positive and -negative cells (corresponding to neurons and non-neurons, respectively) landed on opposite sides of 100 μm^2^ (111 μm^2^ vs. 96 μm^2^), we used a 100 μm^2^ soma area threshold to separate IO neurons from IO non-neurons in P40 animals (Figure 2B3). As astrocytes comprise the largest population of “non-neuronal” cells in the IO, we will refer to the non-neurons as “astrocytes” in the following text.

Next, we proceeded to examine the specificity of the viral constructs for IO neurons over astrocytes. As seen in the examples depicted in Figures 2C,D1, different constructs showed variable preference for large and small cells within the IO, while the expression driven by AAV-PHP.S-CAG was less selective (Figures 2C2,D3). The distributions of cell body areas labeled with the AAV-PHP.S-CAG were always clearly bi-modal with a break at 100 μm^2^ (Figures 2C3,D3) as expected from the immunohistological results shown above. Thus, to quantify the relative neuronal specificity of a given construct, the number of labeled “neurons” (n = 802 across conditions) and “astrocytes” (n = 398 across conditions) in each slice was divided by the numbers obtained with the control virus to obtain an overall “neuron specificity” measure for each construct. Out of the 10 constructs examined this way, we found that 4 clones using Htr5b, Susd4 and Pdx1-promoters could drive stronger transgene expression in neurons than in non-neurons (datapoints to the right of the dashed line in Figure 2E; note that the values shown are calculated from distributions like shown in C3 and D3 but for the entire population). The longest version of the Htr5b-promoter that we previously judged as having the strongest tropism for IO over the surrounding structures (Figure 1) also resulted in the cleanest neuron-specificity (neuron/astrocyte ratio for Htr5b(3.7) promoter: +40 ± 9.8%, n = 79 cells; for all values, see Table 1). Notably, even though Susd4, Pdx1 and Igsf9-promoters have been shown to preferentially label IO neurons when used in the form of a transgenic animal (Hansen and Walmod, 2013; González-Calvo et al., 2021), this was not observed using AAV9-based transfection. Susd4 and Pdx1-controlled expression was only weakly biased toward neurons, and the neuronal expression obtained with various clones of Igsf9 promoter were same or less than that in astrocytes in the same slices. This demonstrates that caution should be used when adapting viral transfection approaches based on results from transgenic animal studies.

Finally, to assess the expression strength of the constructs in IO neurons, the fluorescence intensity in IO neurons driven by the test constructs was compared to that obtained with the control virus in the same cells (Figure 2F). Generally, the results were in line with the relative expression preference for neurons and non-neurons, so that the expression obtained with the long version of Htr5b-promoter was nearly 20 % higher than with the AAV-PHP.S-CAG-tdTomato (+19.8 +/- 5.6%, n = 79; for all values, see Table 2). The promoters with weaker preference for IO neurons over non-neurons (Susd4, Pdx1) also drove lower relative expression in neurons. Intriguingly and building on the observations of the difference in results obtained with AAV-CAG and AAV.PHP.S vectors regarding mesoscale selectivity for IO, the AAV9-construct with the artificial CAG promoter was dramatically biased toward astrocytes (Figures 2D1–D3,E). However, in the few neurons labeled with the AAV9-CAG construct, the fluorescence intensity was stronger than with the AAV.PHP.S-CAG. Thus, despite the non-specificity for IO neurons, the AAV9-CAG-construct can be useful for, e.g., cellular morphometry.

### 3.3. Using Variants of AAV9 and tTA/TRE Enhancer System to Modulate Transgene Expression Density in the IO

As mentioned above, EGFP expression under the nonspecific, artificial CAG promoter was different when using the AAV9 or AAV.PHP.S capsids (Figures 1A3, 2D). This led us to explore how capsid variation would affect expression of the “best” IO promoter identified by mesoscopic IO preference and selectivity for neurons over non-neurons, the Htr5b(3.7). In addition to the AAV9 and AAV.PHP.S capsids, we constructed vectors using the AAV.PHP.eB capsid (Chan et al., 2017; Challis et al., 2019). Furthermore, in anticipation of the need to express transgenes with genetic sequence longer than GFP (such as GCaMP6s) in combination with the long-form Htr5b promoter, we also constructed a cohort of complementary vector pairs taking advantage of the tTA/TRE expression system (Chtarto et al., 2003). Three-color fluorescence expression controlled by the Htr5b promoter in AAV9, PHP.S and PHP.eB capsids was induced by injecting a mixture of AAV9-Htr5b-EGFP, AAV.PHP.S-Htr5b-tdTomato and AAV.PHP.eB.mTagBFP2 (1:1:1 ratio; Figure 3) in 4 animals. In contrast to what we observed when comparing AAV9-CAG and PHP.S-CAG, fluorescence expression under Htr5b(3.7) promoter was significantly weaker with PHP.S than with AAV9 (compare expression shown in Figures 3A1,A2). The vectors with AAV.PHP.eB-capsid drove comparable fluorophore expression to the AAV9 (compare examples in Figures 3A1,A3).

These different expression profiles were maintained and even enhanced in the complementary experiment utilizing three pairs of tTA/TRE-enhancer-drivers of GCaMP6s under Htr5b(3.7)-control (see the examples shown in Figures 3B2,B3). Pooling single-soma intensity measurements obtained for each construct and examining their distributions, it further became evident that with PHP.S-Htr5b-tdTomato and PHP.S-Htr5b-tTA/TRE-GCaMP6s approaches, only small number of IO neurons were labeled while with the AAV9 and PHP.eB capsidss the expression strength varied more smoothly between the extremes (Figures 3C1–C3; see comparison of mean soma intensities for the three capsids in Figure 3D; note that the lower fluorescence levels with PHP.S than with AAV9 or PHP.eB). Furthermore, in PHP.S-transfected IO, the most strongly labeled neurons were located sparsely, as evidenced by the skewed distributions of inter-neuron distances (average distance to closest neighbours 38.1 ± 2, 64 ± 9 and 37.4 ± 2 μm for AAV9, PHP.S and PHP.eB capsids with the tTA/TRE-GCaMP6s vectors, respectively; (1-way ANOVA, [AAV9 vs. AAV.PHP.S] f = 14.5, p < 0.001; [AAV9 vs. AAV.PHP.eB] f = 0.3, p = 0.86; [AAV.PHP.S vs. AAV.PHP.eB] f = 13.9, p < 0.001); Figures 3E1,E2). In summary, out of the 10 tested constructs, we identified the long form of Htr5b as a promising candidate for driving transgene expression specifically in the IO neurons, and noted that packaging the plasmid into different capsids (AAV9, AAV.PHP.S, or AAV.PHP.eB) can bring additional subtlety to the expression profile in IO cellular components.

### 3.4. Expression of GCaMP6s Calcium Sensor in IO Neurons

Above, we screened 10 viral constructs to identify candidates for selective IO neuron targeting based on (1) their selectivity to for IO over the surrounding brainstem structures and (2) their preferred expression in neurons over astrocytes. The Htr5b promoter was the most promising for constructing viral tools for selective fluorescent monitoring of IO neuron's activity. However, it is to be noted that genetic constructs optimized for high expression strength might not be suitable for physiological monitoring. For example, high expression level of calcium buffers may interfere with normal cell function (Müller et al., 2005; Hildebrand et al., 2009; Matthews and Dietrich, 2015 Denizot et al. (2019); Marchena et al. (2020)). Thus, we continued the investigation using a selection of constructs with a wide range of GCaMP6s expression strengths and labeling density:

1. AAV9-Htr5b(3.7)-GCaMP6s (tTA/TRE double virus mix; data shown in green).
2. AAV.PHP.S-Htr5b(3.7)-GCaMP6s (tTA/TRE double virus mix; data shown in orange).
3. AAV.PHP.eB-Htr5b(3.7)-GCaMP6s (tTA/TRE double virus mix; data shown in blue).
4. AAV9-Htr5b(1.8)-GCaMP6s (data shown in red).
5. AAV9-Susd4(2.4)-GCaMP6s (data shown in purple).

To ground our analysis of physiological function to the insights obtained in the preceding sections, we investigated to which extent the intensity of fluorescence observed with confocal microscopy in fixed IO slices (known to correlate with GCaMP6s expression level, see Berens et al., 2018; Éltes et al., 2019) is informative of the level of whole-field fluorescence intensity in live IO neurons. For this purpose, we prepared acute brain stem slices from animals that had been injected with one of the selected 5 construct combinations driving expression of GCaMP6s. 2 weeks after injection we measured the average fluorescence intensity for IO neuron somata (n = 212; at least 30 somata in 4 slices from at least 2 animals for each of the constructs; see methods) with our *in-vitro* whole-field imaging system. After the recording, the slices were immersion-fixed and comparison image data was acquired with confocal microscopy (example images shown in Figures 4A1–A5; see methods for details of optical equipment and intensity measurement). The two measures, live-fluorescence (Figure 4B1) and fixed-fluorescence (Figure 4B2) varied between the constructs and were strongly correlated (R = 0.99, p < 0.01; Figure 4B3).

In line with the results described in previous sections (Figures 2, 3), the strongest overall fluorescence level was obtained with the dual-PHP.eB-virus approach driving GCaMP6s expression under the control of the long-form Htr5b promoter, and the weakest constructs were the single-AAV9 vectors with Susd4 and the short-form of Htr5b promoter (see cumulative plots of individual soma intensities in Figure 4C1). Interestingly, when the soma fluorescence intensity values were normalized to the maximal expression value seen in each slice, the distributions became nearly identical across all of the 5 constructs (K-S; s <0.3, p>0.05 for the 5 conditions) with near-normally distributed values (Figure 4C2; 0.67 ± 0.021%, n = 16 slices; k^2^>0.5, p>0.05 for all distributions). No somata with normalized expression lower than 0.3 were seen in any of the 5 constructs investigated in this manner, suggesting that the background noise levels were similar with no major differences in GCaMP6s expression mechanisms between the single and dual-AAV approaches.

### 3.5. *In-vitro* Calcium Imaging Signatures of IO Spikes Revealed Calcium Signals With GCaMP6s Expressed at Various Concentrations

The observation of intensity distribution normality (Figures 4C1–C2) demonstrates that the GCaMP6s fluorescence level was not saturated in any of the 5 investigated constructs at the selected transfection period (2 weeks). Next, knowing that the average GCaMP6s expression levels differed between the constructs, we investigated how concentration of the probe affects imaging signals. For this purpose, GCaMP6s signals were recorded in acute IO slices (see methods and diagrams in Figure 5) obtained from animals injected with each of the 5 constructs. As shown in example depicted in Figure 5A, calcium events (eCas) could be readily seen in IO neuron somata with 1-photon imaging setup 2 weeks after injection (Figure 5A; 60x objective; 30fps; see methods for imaging parameters). Average waveforms extracted from the 5 constructs are shown baseline-aligned in Figure 5A (see methods for details of soma definition, calcium event detection criteria, as well as fluorescent signal scaling into fW).

Not surprisingly, eCa amplitudes seen with the tTA/TRE-expression constructs were much larger in absolute terms and had up to 20x broader ranges than what was seen using single-AAV9 transfection (F_peak_ 0–200 and 0–10 fW for tTA/TRE and single-virus constructs, respectively; Figures 5A2,A3). On the other hand, the absolute peak intensity values obtained with the three tTA/TRE expression systems were similar (average of F_peak_, 66.6 ± 19, 74.1 ± 23.7 and 45.5 ± 5.5 fW max F_peak_, AAV.PHP.eB, AAV.PHP.S, and AAV9, respectively; Figure 5A3) despite the strongest tTA/TRE construct [PHP.eB-Htr5b(3.7)] having higher baseline fluorescence intensity (F_0_; Figure 4B1). When viewing eCas normalized to the baseline fluorescence intensity (F_0_; compare blue data with red and purple in Figures 5B1–B3), the effect of expression level on calcium signal quality becomes clear: the intermediate GCaMP6s concentrations (corresponding to average F_0_ values between ~ 200 and ~ 1000fW; orange and green data in Figures 5A4,B4) result in best signal-to-noise ratio (SNR) for spike detection. The eCa waveforms obtained with constructs with intermediate expression levels were nearly identical (see peak-normalized average events in Figure 5C1, rise time distributions in Figures 5C2,B3 and relation between eCa amplitude and rise time in Figure 5C4; r = 0.43, p < 0.001). The weakest [AAV9.Htr5b(1.8)] and strongest (AAV.PHP.eB-Htr5b(3.7)-tTA/TRE) constructs showed slight tendency for difference (Figures 5C2,C3) again highlighting the importance of appropriate adjustment of calcium probe concentration as it may influence signal kinetics in addition to the amount of signal saturation.

### 3.6. Spike Activity in GCaMP6s-Expressing IO Neurons

**Figure 6:**
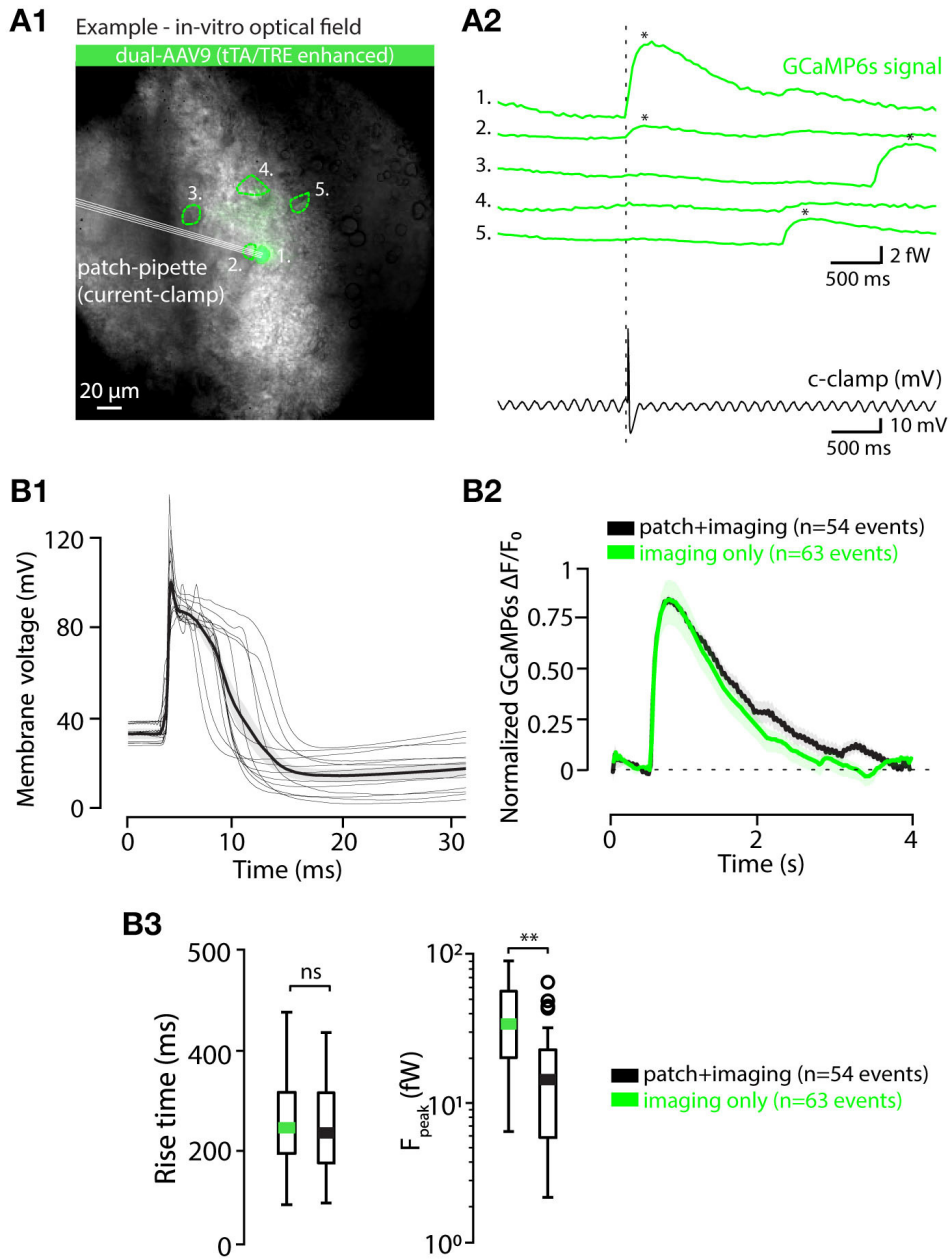
Calcium event waveforms do not differ between patched and intact neurons. **(A)** Example of a combined *in vitro* patch-imaging experiment. **(A1)** shows an infrared-contrast image overlaid with standard deviation projection of fluorescence recording time series, and 5 somata identified in the field of view are indicated with dashed lines (ROIs). Cell labeled 1 was recorded with a patch-clamp pipette. **(A2)** shows the GCaMP6s signals obtained from all of the 5 cells in the field of view (green traces, ROIs indicated with numbers), and the time-aligned electrical recording from cell 1 (black trace). **(B)** Comparison of calcium event waveforms in patched (black) cells and intact (green cells). **(B1)** Time-aligned action potential waveforms from all 14 patched cells, aligned at calcium-event onsets. Thick black trace is the average of all spikes. B2, averaged, onset-aligned and normalized calcium event waveforms peak with shaded areas denoting ± SEM. **(B2)** Comparison between rise times (left) and peak amplitudes (right) of calcium events recorded in patched and intact cells. Green and black bars in box plots represent averages; circles represent outliers. Statistics for eCa event Rise-time between patched and non-patched neurons on **(B3)** (1-way ANOVA, f = 0.085, p = 0.77). Statistics for eCa event peak amplitude between patched (n = 46 events on 13 neurons) and non-patched neurons on **(B3)** (1-way ANOVA, f = 11.09, p = 0.0.0012).

To gain further insights into the capabilities of AAV9-Htr5b(3.7)tTA/TRE-vectors, we acquired whole-cell patch-clamp recordings simultaneously with the *in-vitro* calcium imaging as described above (Figure 6A1; see methods for recording details). As depicted in Figure 6A2 for an example recording from one slice, spontaneous action potentials were seen in current-clamp recording mode, time-locked to the detected calcium events (n = 46 events, 13 cells, 4 animals; Figure 6B1 shows V_*m*_ traces time-aligned to all of the calcium events detected in all experiments). Importantly, the eCa waveforms in IO cells that underwent patching and those that were recorded intact were nearly identical in terms of rise time (Figures 6B1–B3; RT 259.3 ± 16.6 ms and 253.14 ± 13.2 ms for patched and intact respectively; 1-way ANOVA, f = 0.084, p = 0.77 with n = 46 events in patched and n = 64 events in non patched IO neurons) even though the eCa peak amplitudes were smaller in patched cells (when all events were collected from patched-cells, Figure 6B3), due to dilution of GCaMP6s when the cell cytosol is perfused with the patch pipette filling solution (1-way ANOVA, f = 11.1, p < 0.01). To avoid artifacts caused by the progressive washout of GCaMP6s we limited the paired recording duration to about 2 min.

**Figure 7:**
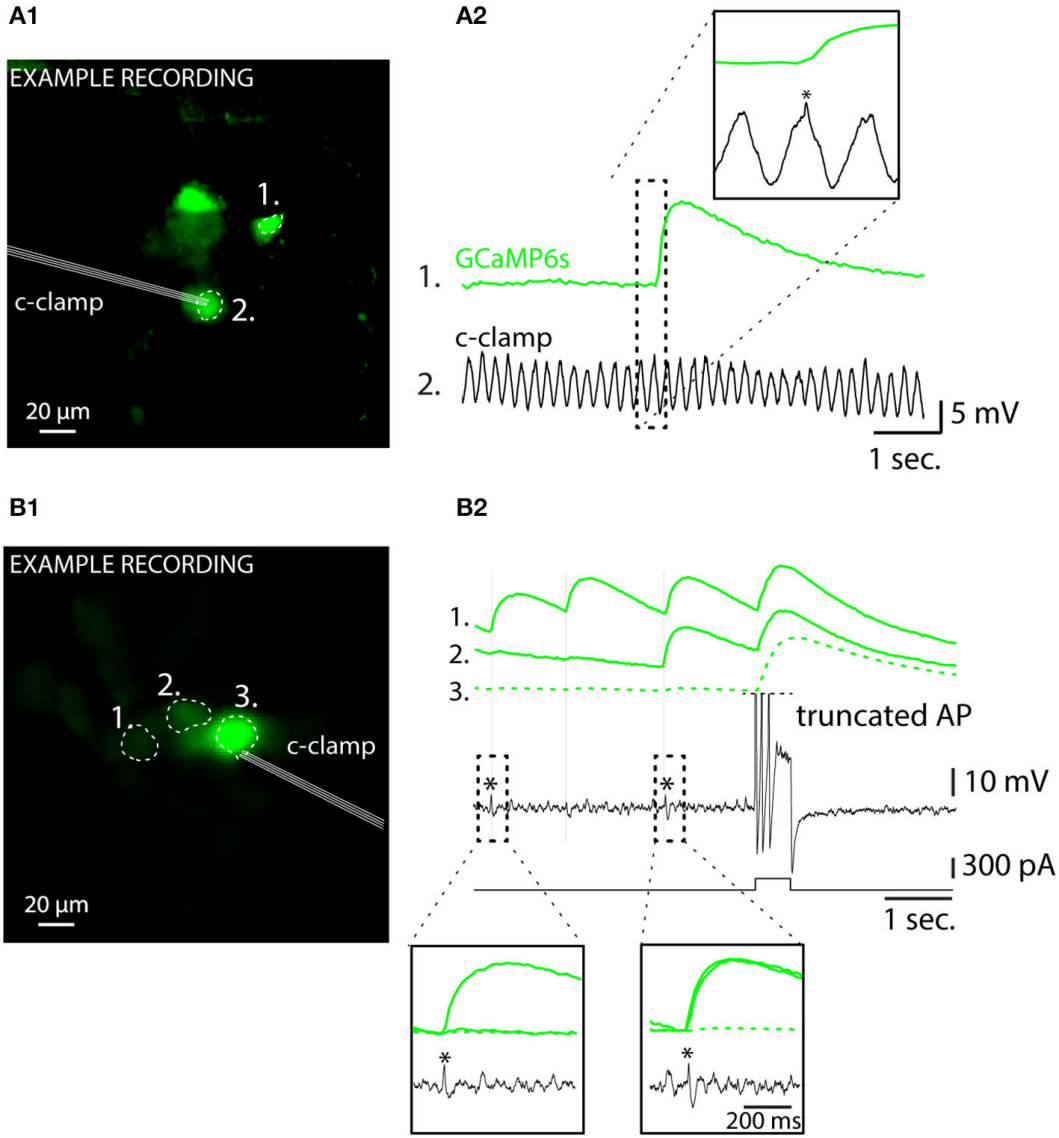
Somatic calcium events are only generated by action potentials occurring in the recorded cells regardless of oscillatory status. **(A)** Simultaneous current-clamp and GCaMP6s imaging shows that a spike in a neighboring IO neuron **(A1)** is reflected as an electrophysiological “spikelet” in the oscillating cell labeled with an asterisk. The image in **(A1)** is a standard deviation time series projection of fluorescence time-series recording. Note that the image brightness is adjusted for viewing and should not be considered as representative of the dynamic range during live imaging. Only cells that spiked during the recording are visible. **(A2)** shows time-aligned fluorescence (top) and V_*m*_ (bottom) traces, and the neighbor-spike-related spikelet in patched cell is indicated with an asterisk in the inset. **(B)** Another example of a simultaneous GCaMP6s and current-clamp recording from 3 IO neurons demonstrating presence of electrophysiological “spikelets” in a patched cell [labeled with “*” in **(B1)**] linked with neighboring cell calcium events. There are three spikelets in the recording, indicated by thin vertical lines, and two of them are enlarged in insets. Current-injection-evoked spiking in the patched cell results in calcium spikes in the neighbor cells, indicative of gap junction coupling.

Among the unique properties of the IO is the strong the electrical coupling *via* gap junctions distributed along tortuously entangled dendritic meshes (Van Der Giessen et al., 2008; Vrieler et al., 2019). This could possibly generate “false-positive eCas” from action potentials that in fact occur in neighboring cell, rather than in the investigated cell, and degrade the fidelity of spike detection. As it is known that action potentials indeed cause small voltage fluctuations (“spikelets;” Lefler et al., 2020) in GJ-coupled neurons, they might be possibly associated with measurable somatic calcium fluctuations. However, none of the detected spikelets were accompanied by detectable eCas in the 13 patch-clamped IO cells (Figures 7A,B; (A2,B2) show examples from oscillating and non-oscillating cells), even though they could be often linked with an eCa in one or more nearby somata (Figures 7B1,B2). Thus, we conclude that eCa signals allow reliable localization of IO action potentials even in case of synchronous activity in the GJ-coupled neighborhood.

In the following sections, we present further proof-of-concept results obtained with the new constructs, suggesting avenues for further work. It should be stressed, though, that the suitability of the tool must be specifically considered in the context of each question examined, as the possibility of interactions between the calcium-binding probe and cellular physiology cannot be excluded.

### 3.7. Variability of GCaMP6s Event Waveforms and Action Potential Shapes

**Figure 8:**
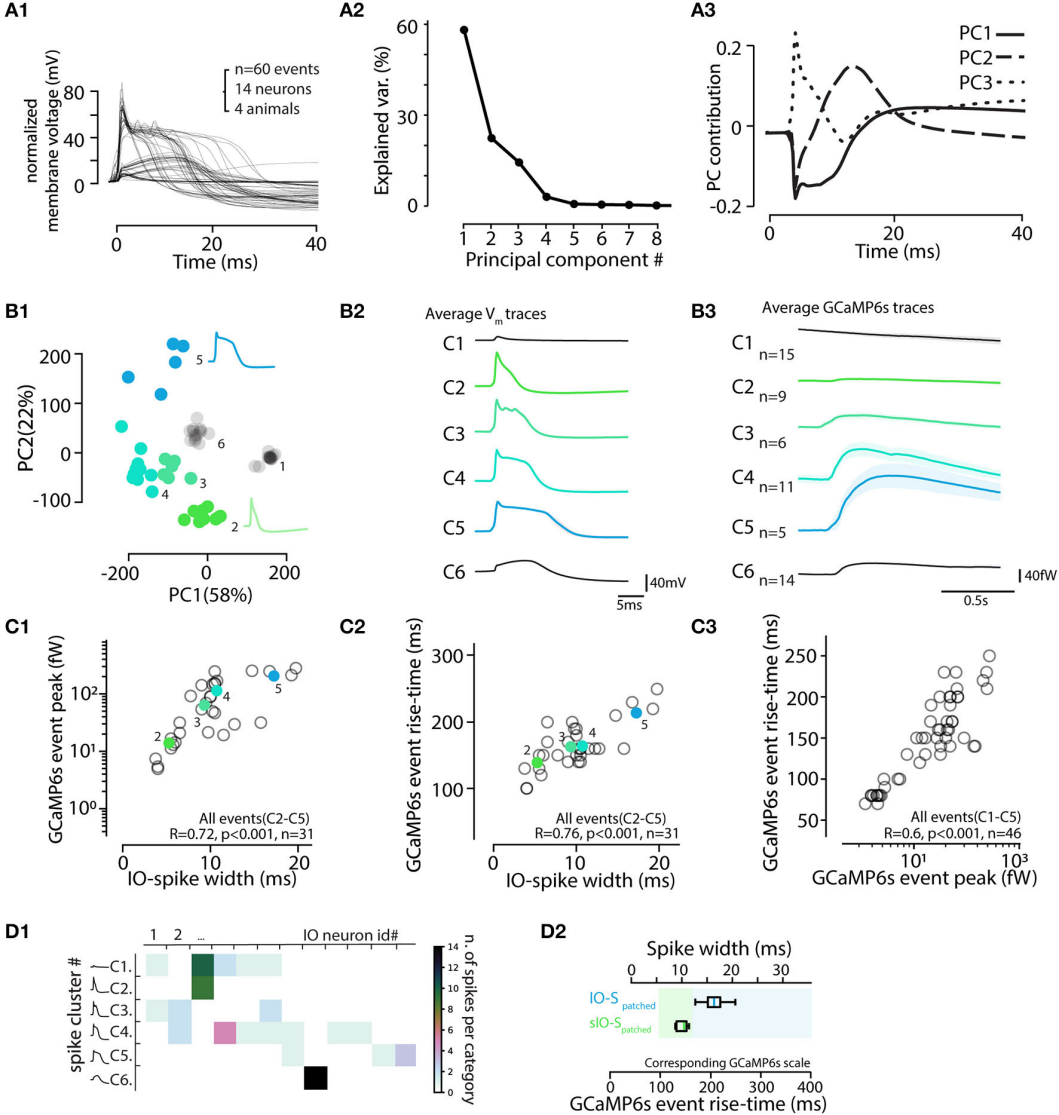
Electrophysiological features of IO action potentials are reflected in the GCaMP6s event waveform. **(A)** Principal component analysis of IO action potential waveforms. **(A1)** 60 voltage spike events detected and aligned on initiation point. **(A2)** Relative contributions of the principal components to the waveform variability. The 3 first PCAs explain 95%. **(A3)** Time-varying contributions of the 3 first PCAs, corresponding to the “calcium shoulder” (solid line), after-hyperpolarizarion (dashed line), and the sodium spike (dotted line). **(B)** Clustering IO calcium events (eCAs) based on the PCA analysis in **(A)**. **(B1)** *k*-means clustering (indicated by colored markers) of the IO action potentials shown in **(A1)**. **(B2)** Averaged electrophysiological waveforms from the clusters identified in **(B1)**. **(B3)** The average calcium event waveforms linked to the electrophysiological clusters in **(B1,B2)**. Note that no noticeable calcium transients are seen linked with cluster 1 that corresponds to “spikelet” events (see Figure 7). Also, the calcium events belonging liked to cluster 6 events lacking a full sodium spike are very small in amplitude. Spikes from cluster 6 are excluded from rest of analysis, and only the 46 spikes [corresponding to clusters 1–5] recorded in 13 healthy IO neurons are depicted in following. **(C)** Calcium event peak amplitudes **(C1)** and rise times **(C2)** strongly correlate with the electrophysiological spike widths (n = 31 events, clusters 2–5). Note that the eCa amplitude-to-spike width relation saturates with longest spikes, while rise time to spike width relation is more robust. **(C3)** Relation between calcium event rise time and peak amplitude. Note slight non-linearity with largest events. The colored points and black circles represent average values for respective clusters and individual observations respectively. Events from clusters 1 and 6 are not shown in **(C1,C2)** as they correspond to spikelets and unhealthy neuron spikes. **(C3)** consists of data from clusters 1–5. **(D)** Spike cluster diversity in different recordings. **(D1)** Number of spike cluster observations for each cell. Note that the “incomplete spikes” (cluster 6) and “short IO spikes” (cluster 2) are only seen in single cells each while spikes from other clusters are seen in multiple cells. **(D2)** Box-plot including data from clusters 2–5 providing suggestive translation scale between observed calcium event rise time (bottom scale) and the corresponding electrophysiological spike width (top scale). Calcium events with rise times longer than 150 ms correspond to “normal” IO spikes (IO-S). The “short IO spikes” (sIO-s) do not present a clear calcium shoulder and may be caused by unusual physiological state of the IO neuron, possibly due to major damage to dendrites caused by slice preparation.

Neuron action potentials in brain regions other than the IO have remarkably invariant waveform and short duration (less than 1 ms) and thus little if any variation of action potential shapes is expected to be reported by calcium imaging sensors. However, the IO neuron action potentials are characterized by a highly variable post-spike calcium plateau and prolonged depolarization (“calcium shoulder”) that can last up to 20 ms or more (Gutnick and Yarom, 1989; Chorev et al., 2007a; Mathy et al., 2009. For visualization of the characteristic components of the IO spikes, we decomposed the patch-clamp-recorded waveforms (Figure 8A1) into their principal components. As shown in Figures 8A2–A3, the 2 first components corresponding to “calcium shoulder” and “afterhyperpolarization” explained almost 80% of the variability.

Next, we examined if the information about IO spike waveform could be decoded from the shapes of the corresponding GCaMP6s transients, despite the orders-of-magnitude difference in the IO spike and eCa duration. To facilitate analysis and provide insights beyond correlations, Figure 8B1 shows all of the 60 recorded electrophysiological events in the space of the two first PCAs and the results of k-means clustering (k = 6 chosen by “elbow method” from sum-of-square curve; see methods for details). In the present dataset (n = 60 events from 14 cells recorded in 10 brainstem slices of 4 animals), each identified cluster contained 5 to 15 spikes. The averaged waveforms of each electrophysiological cluster are shown in Figure 8B2, and the averaged eCas for the same clusters are in Figure 8B3. In line with the observations shown in Figure 7, the calcium transients corresponding to “spikelet” events forming cluster 1 were neglible, while the “full-blown” action potentials were linked with eCas of varying sizes. Notably, in some IO neurons, all of the electrophysiologically recorded action potentials lacked a clear sodium spike component (cluster 6). These cells likely represent IO neurons that have their axons on a dendrite that might have been damaged during slicing (Ruigrok et al., 1990), thus preventing the fast sodium spike from being fully formed. This is also evident in the eCas for this cluster (Figure 8B3) that only had a small and slow calcium transient. Spikes from cluster 6 were excluded from the rest of the analysis. As depicted in Figure 8D1, spike shapes belonging to clusters 1, 3, 4, and 5 were all found in multiple cells.

Combining data from the 45 spikes recorded simultaneously with GCaMP6s and patch-clamp recording, we saw a strong correlation between the width of the electrophysiologically recorded IO spike and eCa amplitude and rise-time (Figures 8C1,C2). As expected from the possible GCaMP6s signal saturation with highest-magnitude calcium influxes, the correlation between spike widths and their eCa peak amplitudes was reduced in slope for cluster). However, rise-time was translating the action potential width with more fidelity (R = 0.076, p < 0.001). Together with the nearly-linear relation between calcium event amplitudes and rise times (Figure 8C3), the results suggest that a considerable fraction of the variability in the electrophysiological IO spike duration could be read out in the GCaMP6s event shapes (Figure 8D2). Furthermore, IO spikes retain the variable waveforms despite the activity of a calcium probe.

### 3.8. Calcium Imaging of IO STOs

As mentioned in the introduction, the IO neurons express two unusual calcium-influx-related electrophysiological features: the extended “calcium shoulders” of IO spikes and the subthreshold oscillations (STOs) of membrane voltage that do not usually exceed 10 Hz in frequency. After confirming that the calcium event features recorded with GCaMP6s expressed at intermediate concentrations could provide information about the IO spike calcium shoulders, we wondered if it would be feasible to observe IOn STOs as well.

**Figure 9:**
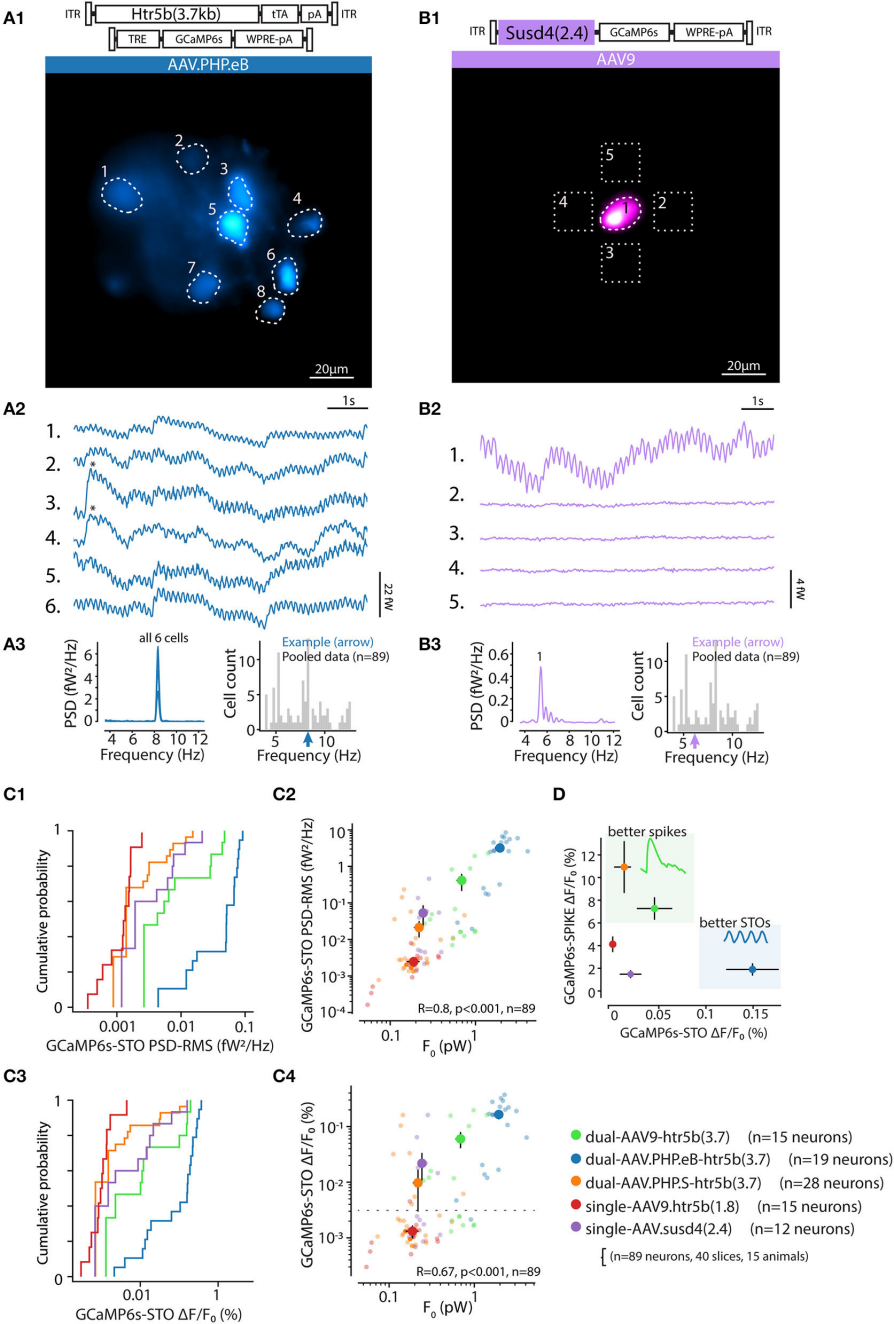
*In-vitro* calcium imaging of IO subthreshold oscillations. **(A,B)** Example *in vitro* calcium imaging recordings from dense **(A)** and sparse **(B)** transfection, using the constructs as indicated above the panels. Identified IO neuron somata are indicated with dotted ROIs in time-series projection images in **(A1,B1)**; note that the image brightness is adjusted for viewing and should not be considered as representative of the dynamic range during live imaging. The GCaMP6s traces corresponding to labeled ROIs are shown in **(A2,B2)**. In the example shown in **(B)**, only one cell is labeled, and 4 neighboring regions indicated by dotted squares in **(B1)**, are shown as ROIs representing signal from non-transfected cells. Asterisks in **(A2)** indicate detected calcium spikes. Note the different vertical scales in **(A2,B2)**, reflecting lower GCaMP6s concentration in **(B)**. **(A3,B3)**: Welch spectra for the example recordings show a sharply defined frequency peak for the oscillating cells (left). Right show the peak frequency of the example recordings (colored arrows) with respect to the entire population (gray bars) **(C)** The power of observed oscillations in calcium traces is stronger with higher baseline fluorescence level resulting from higher concentration of GCaMP6s. Colored lines [in **C1,C3**] and markers **(C2,C4)** represent data obtained with the 5 different constructs as indicated at the bottom right corner of the figure. **(C1,C2)** display the ranges of oscillation in terms of STO-bandwith power **(C1)** and oscillation peak-to-peak amplitude **(C2)**. Note that the oscillation amplitude increase saturates below 1% **(C3)**, while all of the constructs were shown to report spike amplitudes with several-fold higher (Figure 4). **(C2,C4)** display the relation between single-cell baseline fluorescence intensity (F_0_, *x*-axis) and oscillation power **(C2)** and peak-to-peak amplitude **(C4)**. Dotted horizontal line in **(C4)** represents the threshold of 0.12 % DFF for classification of the signal as “oscillating” as determined in Figure 10. **(D)** Summary of imaging capabilities of the different constructs for reporters of IO action potentials (vertical axis) and STOs (horizontal axis). The constructs with intermediate expression levels (orange and green data points; see Figure 4B) provide better resolution of spike waveforms. The constructs with highest expression (blue data) under-perform with spike amplitudes but provide high resolution of the subthreshold oscillations.

Indeed, in most of the acute slices prepared from animals transfected with the 5 constructs for live imaging, we observed sinusoidal fluctuations of the GCaMP6s signal. As shown in the examples presented in Figure 9 (A and B, from densely and sparsely-labeled IO, respectively), the oscillations could be seen in numerous somata, with peak frequencies (ranging between 3.5–13 Hz) closely matching within one field of view as expected for the subthreshold oscillations in IO cells (Figures 9A3,B3; n = 89 cells in 40 slices, 15 animals); (Long et al., 2002; Lefler et al., 2014).

We further observed that the clarity of GCaMP6s STO signal was directly related to the concentration of GCaMP6s in a given IO neuron, so that the best SNR were obtained with the highest GCaMP6s concentrations (resulting in high F_0_ values). STO signal was clearest when using the AAV-PHPe.B-Htr5b(3.7)(tTA/TRE) (blue data in Figures 9C1–C4; *x*-axes indicate strength of oscillation in STO range). This was evident in the distribution of both oscillation amplitude measured as in the power of the calcium signal in the STO range (Figures 9C1,C2) and as well as maximal DFF in the band-passed calcium signal (Figures 9C3,C4). The resolution of calcium fluctuations was not limited by signal saturation as the highest DFF values reached were nearly an order of magnitude lower than those recorded from IO spikes in the same cells (Figure 9D).

**Figure 10:**
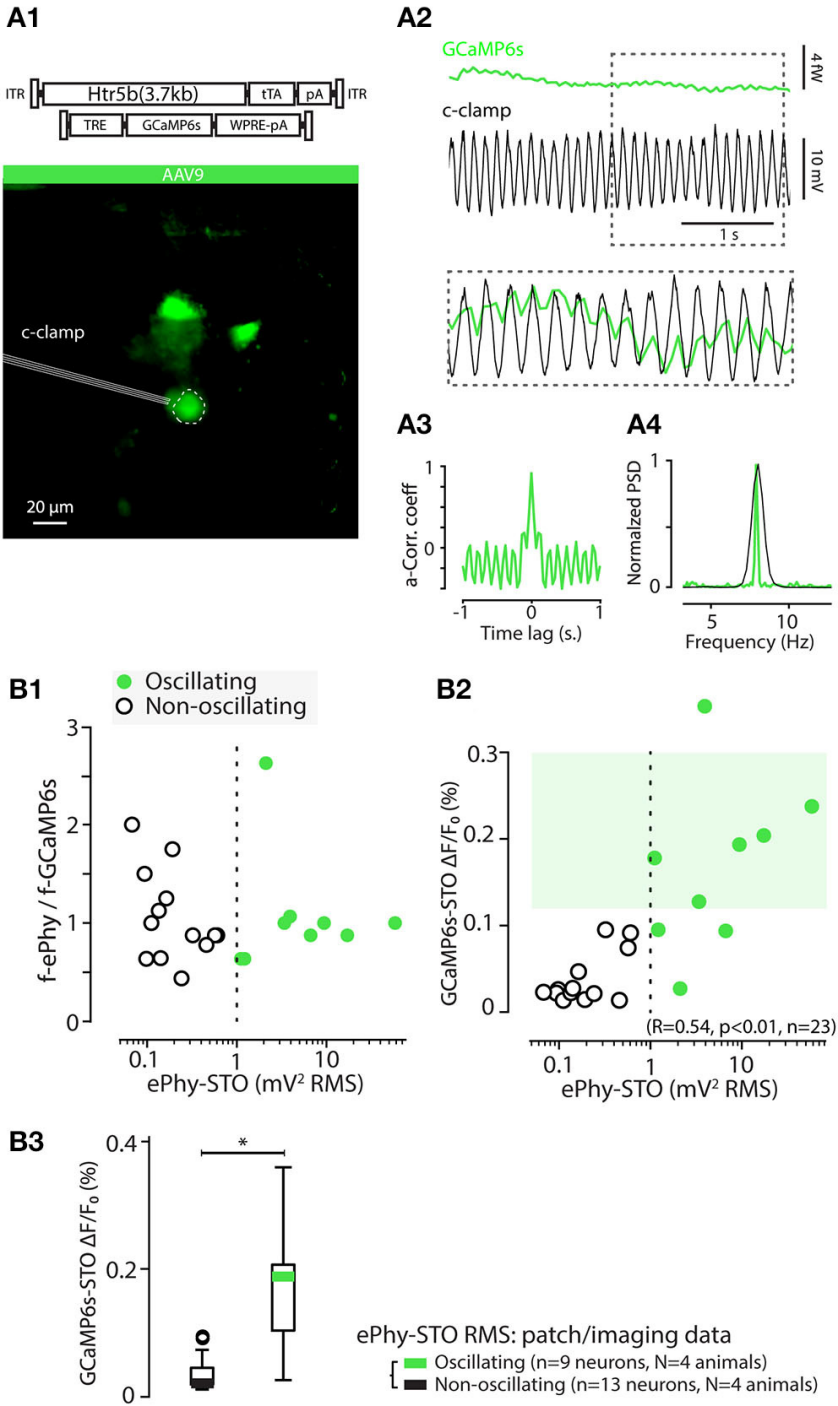
GCaMP6s-STOs are reporting electrophysiological STOs *in vitro*. **(A)** Example of a simultaneous patch clamp and calcium imaging recording of an IO cell *in-vitro*. **(A1)** shows the standard deviation projection image of the recorded time series, with the patched cell indicated with a dashed line. Note that the image brightness is adjusted for viewing and should not be considered as representative of the dynamic range during live imaging. **(A2)** time-aligned GCaMP6s trace (top) and (V_*m*_) trace (middle). The section of traces indicated by dotted rectangle is shown overlaid in bottom. The autocorrelation of the calcium trace **(A3)** as well as comparison of its Welch spectrum with that of the electrophysiological recording **(A4)** show stable oscillatory behavior in this cell. **(B)** The frequency of GCaMP6s STOs closely matches that of electrophysiological recordings. **(B1)** Frequency ratios of voltage (*y*-axis) and fluorescence (*x*-axis) signals is flat over large range of oscillation power. Dotted line indicates a threshold of 1 mV^2^ RMS used to classify cells as “oscillating” or “non-oscillating.” **(B2)** Relation between electrophysiological STO power and GCaMP6s STO amplitude. Dotted line indicates the 1 mV^2^ RMS threshold value as in **(B1)**. **(B3)** Comparison of STO power of the GCaMP6s signal obtained from cells classified as oscillating (green) or non-oscillating (black) based on the 1 mV^2^RMS threshold (Welch t-test, t = -4.99, p < 0.05).

To confirm the common origin of the observed calcium STOs (GCaMP6s-STO) and electrophysiological STOs, we compared simultaneous current-clamp and GCaMP6s imaging experiments conducted in the acute IO slices. In the 22 electrophysiological recordings included in the present study, roughly half of the cells displayed easily-identifiable electrophysiological STOs. As shown for the example recording (Figures 10A1,A2), voltage STOs could be neatly superimposed with the calcium fluctuations and their peak frequencies matched precisely, even though the higher temporal resolution of patch-clamp recording provides much finer resolution of the frequencies (Figures 10A3,A4).

The amplitude of the GCaMP6s-STO correlated strongly with the power of the electrophysiological STOs (Figures 10B2,B3). The GCaMP6s-STO amplitude was very low in non-oscillating somata (defined by STO power in V_*m*_ lower than 1 mV^2^RMS). Furthermore, the GCaMP6s-STO peak frequencies closely matched the electrophysiological STOs recorded simultaneously (Figure 10B1) when the cells were displaying STOs (right part of dashed line). A 0.12 % DFF threshold was calculated from the 5th percentile of the oscillating neurons (upper light green square), is such a way non-patched neurons displaying STO-PSD above that value can be considered as oscillators. It should be mentioned that it is possible that at the recording frame rate of 30 Hz, highest STO frequencies are not faithfully represented. Nevertheless, the IO STO frequencies should be well below the theoretical limit (15 Hz) based on sampling theory. Thus, we conclude that presence of calcium fluctuations with GCaMP6s-STO amplitudes larger than 0.1% DFF (corresponding to 0.00024 fW2/Hz RMS) is a signature of electrophysiological STOs with similar frequency range. However, to reach such high GCaMP6s-STO DFF for the low-amplitude STO, a high expression level of the GCaMP6s is needed, leading to lower SNR in resolving action potentials (Figure 6).

### 3.9. *In-vivo* Imaging of IO Spikes and STOs

Above, we show that GCaMP6s expression driven by the Htr5b promoter in adeno-associated viral constructs can be used to monitor the entire range of IO neurons' unique electrophysiological features from action potential waveforms to subthreshold oscillations. Here, we provide proof-of-concept results demonstrating that the approach can also be used for the more demanding conditions of *in-vivo* experimentation.

**Figure 11:**
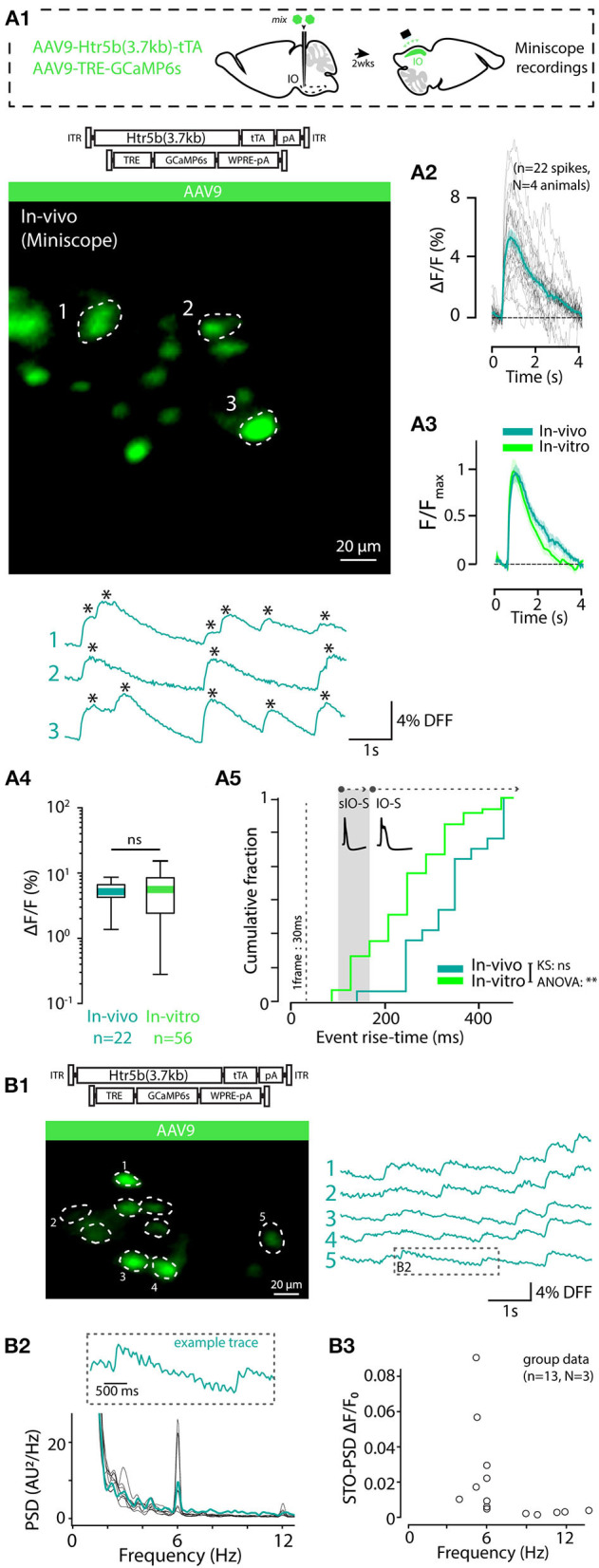
Imaging IO spikes and STOs *in vivo*. **(A)** *in-vivo* calcium spike recording from ventral IO using a GRIN-lens-based miniscope. The schematic on top of the figure describes the approach. **(A1)** Example image (standard deviation projection of the time series) from a living IO with several IO somata indicated by dashed-line ROIs and numbered labels. Note that the image brightness is adjusted for viewing and should not be considered as representative of the dynamic range during live imaging. The calcium traces extracted from the labeled ROIs are shown below the image, with detected spikes indicated by asterisks. **(A2)** Detected spikes from pooled N = 4 animals, 22 cells, aligned on the initiation point. Dark green trace is the averaged waveform. **(A3-5)** Comparison of the calcium event waveforms between *in-vivo* (dark green) and *in-vitro* (bright green) recordings. The average amplitudes are not different **(A4)** but the shortest calcium events are absent *in-vivo* recordings **(A5)**. Gray shaded area represents the range of calcium event rise times measured *in-vitro* corresponding to “short-IO-spikes” (sIO-s; cluster 2 in Figure 8B). For reference, the vertical dashed line in A5 indicates duration of a single frame in miniscope recording. **(B1)** Example of oscillating IO neurons recorded *in-vivo*. Eight active cells could be detected (ROIs indicated with dashed white lines; standard deviation projection of the time series) and traces from the labeled cells are displayed on the right. Welch spectra of the 8 ROI are shown superposed in **(B2)**, with one example cell [labeled 5 in **(B1)**] is highlighted in dark green. **(B3)**; Population data of oscillating neurons found in N = 3 animals. Each circle represents a single IO cell.

As the extremely deep location of the IO is prohibitive for conventional cranial window-based imaging approaches, we employed our recently-described method for GRIN-lens based, whole-field miniscope imaging of the ventral IO in an anesthetized mouse (Guo et al., 2021). In anesthetized mice where AAV9-Htr5b(3.7)-tTA/AAV9-TRE-GCaMP6s mix was injected 2–3 weeks earlier, numerous fluorescent IO neuron somata were visible (Figure 11A1) and calcium spikes could be readily extracted from somatic fluorescence traces as was done with acute slice imaging (Figure 11A1, bottom; compare with Figure 5). The spikes were often synchronous across multiple neurons in the field of view, with amplitudes ranging from 1 to 12% DFF (Figure 11A2), and event frequencies and waveforms closely conforming to those recorded with the same construct *in-vitro* (DFF peak amplitudes 4.98 ± 0.39, 7.27 ± 0.0.95%, 1-way ANOVA; f = 1.43, p = 0.24); (rise times 351 ± 23.8 and 253 ± 13.7 ms, 1-way ANOVA; f = 10.2, p < 0.05); for *in vivo* and *in-vitro*, respectively (Figure 11A4). Notably, the *in-vitro* eCa rise time distribution consisted of a range of shorter-duration events when comparing with those recorded *in-vivo* (Figure 11A5), suggesting that the occasional IO spikes with a barely distinguishable calcium shoulder (depicted as an inset in Figure 11A5 and corresponding to cluster 2 in Figure 8) possibly result from slicing-related disturbances in neuronal physiology.

Careful examination of the fluorescent traces extracted from the somata in the *in-vivo* ventral imaging approach revealed that oscillatory fluctuations were seen in well-labeled somata even when using the AAV9-Htr5b(3.7)-tTA/TRE construct that did not deliver the best GCaMP6s-STO resolution in our *in-vitro* experiments (Figures 11B1,B2). Similarly to *in-vitro* experimentation, oscillations were seen in multiple cells in a given field of view and they were tightly matched in frequency (Figure 11B2). As shown in Figure 11B3, GCaMP6s-STO oscillations with power in exceeding the threshold (as determined in Figure 10B2) were recorded in 19 somata in 3 animals. The peak frequencies differed between recordings, and the range of detected frequencies (Figure 11B3) was comparable to our *in-vitro* recordings. Thus, the viral constructs based on the Htr5b promoter and tTA/TRE enhancer system can not only be used to monitor the spike shape variability in the IO but also reveal the presence of STOs *in-vivo*.

### 3.10. *In-vivo* Imaging of Climbing Fiber Activity in Cerebellar Cortex

**Figure 12:**
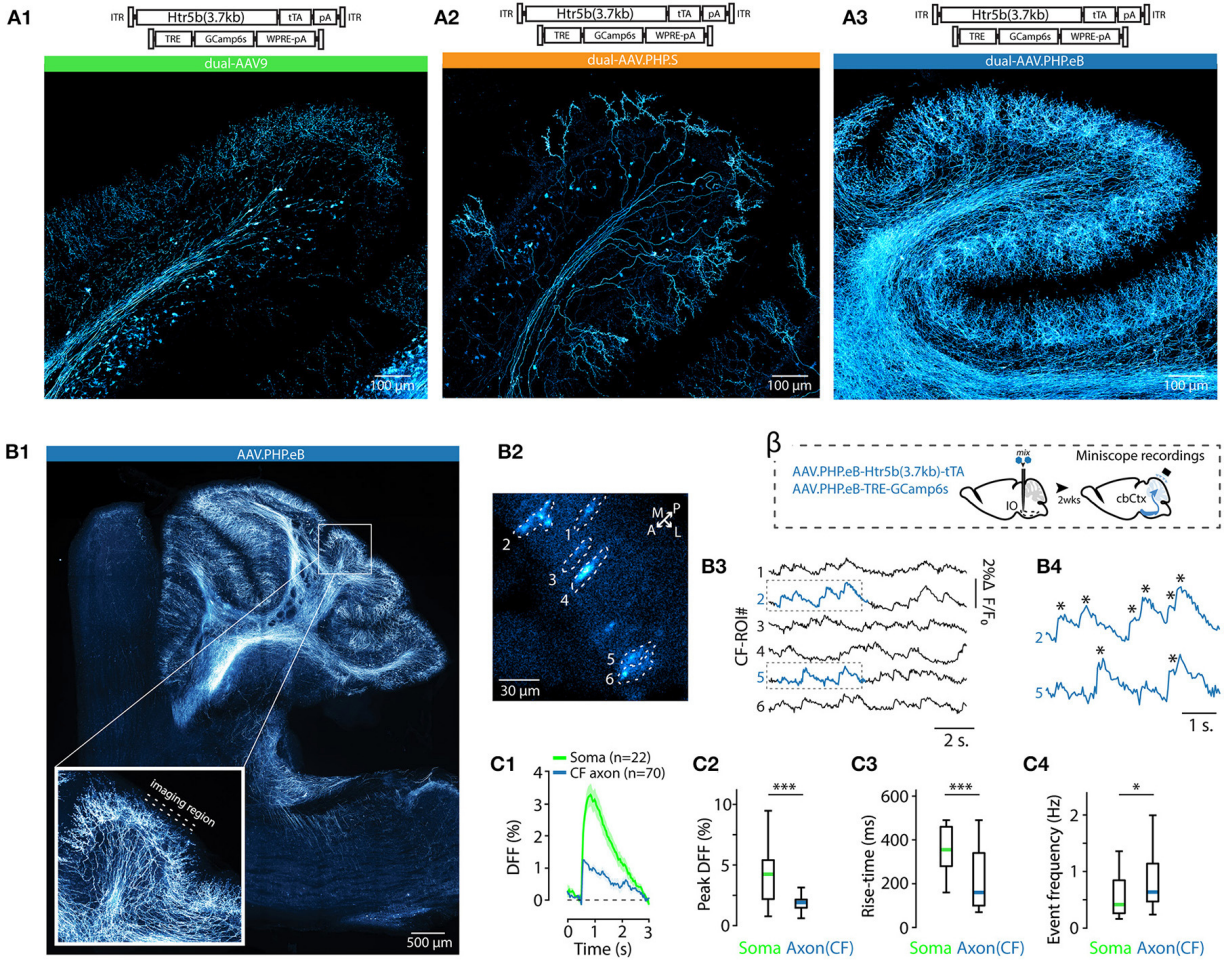
Recording of climbing fiber calcium events in vivo. **(A1–A3)** Expression of GCaMP6s in cerebellar climbing fibers with AAV9, AAV.PHP.S, AAV.PHP.eB-Htr5b(3.7)-tTA/TRE constructs, respectively. **(A1-A3)** are tiled and Z-projected 40x confocal images of sagittal cerebellar sections showing expression of GCaMP6s in axons in the cerebellar cortex with samples prepared for anatomical study. Expression of GCaMP6s in CF is sparse in when the viral serotype is AAV.PHP.S, while dense and widespread with AAV.PHP.eB serotype. The inset [in **(B1)**] indicates the region that was imaged in with the miniscope (see schematic on top of the figure). **(B)** Example recording from CFs in a living mouse. **(B1)** Is a sagittal slice from tissue on the same brain where *in-vivo* calcium imaging was done. A schematic labeled *beta* describes the experiment procedure. **(B2)**, *z*-projection image (standard deviation) of miniscope calcium recording time series (20 second recordings per zone) as seen from the dorsal surface of the cerebellar cortex. Anterio-posterior (AP) and medio-lateral (ML) directions are indicated with white arrows. The dashed lines indicate manually-drawn ROIs for CFs, and the respective calcium traces are shown in **(B3)**. Parts of two of the traces [2 and 5, indicated by dashed rectangles in **(B4)**] are shown with expanded time scale at the bottom of the panel. Detected events are indicated by asterisks. **(C)** Comparing *in vivo* calcium events recorded in IO axons (CFs; blue) with the somata (green). n = 70 events in 2 animals for axon recordings, 22 events in 4 animals for somata. **(C1)** Average *in-vivo* GCaMP6s transients from CFs and IO somata, aligned at initiation point. Shaded regions represent ± SEM. **(C2)** Comparison of calcium event amplitudes in IO somas and axons when normalized to basal fluorescence (1-way ANOVA, f = 8.19, p < 0.001). **(C3)** Comparison of event rise-times between IO soma and climbing fibers (1-way ANOVA, f = 5.3, p < 0.001). **(C4)** Comparison of the instantaneous frequency of events (inverse of inter-event interval) in the axon vs. the soma (1-way ANOVA, f = 5.35, p < 0.023). Note the slower spike rate in somatic recordings, possibly due to tissue cooling during the ventral surgery needed for somatic recordings.

Finally, when examining the brains from animals used for the experiments above, we noticed fluorescent labeling in the axons leaving the IO with the with the some of the constructs (see, e.g., Figures 3A1–A3). This suggested that using this transfection approach, strong GCaMP6s expression could be driven in the cerebellar cortex where the IO axons terminate as climbing fibers (CFs). Indeed, as shown in example confocal images from sagittal sections of the cerebellar cortex (Figures 12A1–A3 where anatomy of CF was visualized in at least 4 animals per construct), CFs were seen clearly labeled with all of the constructs based on Htr5b promoter and tTA/TRE enhancing system. The labeling strength was in line with the expression analysis shown in Figure 3, so that the CFs were seen sparse AAV.PHP.S double-virus approach and the AAV.PHP.eB-approach resulted in labeling the entire extent of IO axon pathway in all regions of the cerebellar cortex (Figure 12A3; a composite image of a slice containing both IO and cerebellar cortex is shown in Figure 12B1). To examine whether the GCaMP6s expression with this construct allows *in-vivo* recording of climbing fiber spiking, we opened an acute cranial window above the cerebellum in an anesthetized mouse (see schematics in a box above Figure 12B) and placed the GRIN-lens of the miniscope on the cerebellar surface (recording location shown for a post-experiment perfusion-fixed cerebellum in Figure 12B1). In both of the 2 animals in which this approach was tried, clear calcium transients could be seen originating in stripe-shaped narrow regions in the cerebellar cortex (Figures 12B2–B4) as expected for CF endings. Notably, the amplitudes of recorded calcium events, as well as rise times were lower than those acquired from the much larger IO somata with the same viral construct (averaged traces from CFs and somatic recordings are shown baseline-aligned Figure 12C1, and their amplitudes and rise times are compared in C2-3). This is expected as the axonal events measured at distal tips do not consist of the “calcium shoulder” (Mathy et al., 2009). The calcium-event frequencies recorded in the IO somata were slightly lower than the ones recorded in the axon (Figure 12C4), likely due to cooling of the IO during the surgical procedure. This difference is possibly underestimated as reliable detection of spikes in the axonal tips would need higher sampling rate. Nevertheless, we show here the potential of the genetically-encoded calcium indicator GCaMP6s in investigating a large range of electrophysiological events *in-vivo*. Importantly, this is feasible only with careful adjustment of the probe expression by means of choosing the right combination of viral capsids, promoters and enhancer systems.

## 4. Discussion

In this work, we describe construction and validation of a viral vector to monitor the subtle activity signatures of inferior olive neurons *in-vitro* and *in-vivo*. By means of systematic and quantitative comparison of expression profiles of various constructs with respect to that of a non-specific vector (PHP.S-CAG), we explored promoter sequences that would show stronger preference for expression in the IO than commonly-used general promoters with broad expression (See Table 1). From a batch of 10 promoters selected from existing literature, we identified a clone of the serotonin receptor subtype 5b promoter [Htr5b(3.7)] as a sequence of interest. Injection of viral constructs driving gene expression under this promoter showed strong selectivity for IO over the surrounding brainstem (Figure 1), as well as virtually exclusive and strong expression in neurons as opposed to astrocytes (Figure 2). Next, we took advantage of the differing expression profiles of three viral capsids (AAV9, AAV.PHP.S, and AAV.PHP.eB; Figures 3, 4) to express the calcium reporter GCaMP6s at various concentrations and utilizing the tTA/TRE expression system to compensate for the limited packaging capability of AAV vectors. With “intermediate” expression levels (AAV9 and AAV-PHP.S), wide-field 1-photon fluorescence imaging reliably resolved IO action potentials and also allowed estimating the width of their “calcium shoulder” component (Figures 5–8). Furthermore, when PHP.eB was used in conjunction with Htr5b(3.7) promoter and the tTA/TRE enhancing system to drive higher expression level, GCaMP6s imaging could be used to monitor the IO subthreshold oscillations (STOs) simultaneously with IO spikes (Figures 9, 10). Finally, the potential of the viral transfection tools for IO imaging were demonstrated in living mouse IO neuron somata and their axons (climbing fibers) by directly imaging the cerebellar cortex with an endoscope.

To our knowledge, the constructs designed in this work are the first fluorescent probes that allow monitoring subthreshold and suprathreshold activity in the IO, both *in-vitro* and *in-vivo*. While the possible physiological side-effects of supplementing a calcium sensor to the intracellular calcium buffering capacity have not been investigated in detail in IO neurons, we hope that the constructs (deposited at Addgene plasmid repository https://www.addgene.org/) will be taken to wide use in the olivo-cerebellar research community. It should be stressed that as in any studies relying on calcium-binding probes, the expression levels must be carefully adjusted to the questions at hand; specifically, signatures of overexpression artifacts, such as the cytomorbid nuclear-filling phenotype, must be understood and eliminated. If over-expression of the probe is a concern, shorter expression times and/or lower viral exposure should be used. For most sensitive experimentation other methods such as voltage imaging (Dorgans et al., 2020) should be used.

Below, we discuss the most important insights rising from our work regarding use of viral tools and importance of careful attention to vector design as it can be used for targeting neuronal populations of different brain structures outside the olivocerebellar system (Gompf et al., 2015; Botterill et al., 2021).

### 4.1. Steps to Develop a Reliable AAV-Based Calcium Imaging Vector

In order to compensate for experimental variability arising from differences between experimental animals, experimenter skills and equipment, care is needed in designing the workflow of transfection and expression assessment. Here, we list our recommendations for future work.

#### 4.1.1. Design of Transfection Experiments

To ensure near-identical sample quality and allow comparison between experiments that possibly have been conducted months apart:

1. Use the same number of AAV particles per injection per mouse and same volume of injectate.
2. Mix the test-construct solution with a well-characterized control construct, using same titer and ratio for all injections.
3. Target the viral injections strictly at the same coordinates, paying great attention to the head and body angle before and after drilling, and use same procedure for injection pipette insertion and removal.
4. Maintain the same number of days as expression time for all experiments.
5. Animal perfusion, brain sectioning and confocal slide preparation procedures must be identical; for best reproducibility, image acquisition should be done within similar time windows from sample preparation to avoid signal degradation.
6. To compensate for other unavoidable variations, use same number of animals for each construct, preferably randomizing injection order to avoid “Monday effects.”

#### 4.1.2. Design of Confocal Image Acquisition and Analysis for Expression Profiling

1. Confocal image acquisition parameters (such as laser power, scanning speed, z-stack depth, emission wavelengths and photomultiplier gains) are to be defined once using a representative sample. Theoretically this should provide comparable intensity signals, assuming that the sample preparation is identical. While this theoretical optimum can not be reached in practice, it is essential to strive to minimize equipment-related variability for the hope of comparability.
2. The post-processing of images (such as cropping, histogram adjustments if done, channel subtractions) should be done automatically and with standardized parameters using image processing software such as FIJI) for all the channels in all experiments. Batch processing (rather than manual adjustments) supports systematic workflows and reproducible results.
3. All extracted measurements of the construct expressions should be planned to be reported in relation to the values obtained with a reference construct, injected at the same time and at same viral count as the test construct (see above). This allows compensating for between-sample variability in viral particle diffusion in the brain. Note that any known non-specific viral vector can be used as the reference construct.

#### 4.1.3. Design of Experiments and Analysis to Assess GCaMP6s Signal Quality

A major issue of calcium imaging experiments using genetically encoded probes (GECI) such as GCaMP6s for imaging neuronal activity is the lack of control of protein expression and the possible interference between GCaMP6s and intrinsic calcium dynamics (Steinmetz et al., 2017; Denizot et al., 2019). If the imaging experiments were conducted with cell-permeant or patch-applied calcium dyes (such as Oregon Green Bapta or other organic dyes; (Paredes et al., 2008)), the effect of the dye concentration on physiological processes could be assessed. Here, we took advantage of the different expression strengths of the constructs leading to different concentrations of GCaMP6s, allowing correlating features of calcium transients with relative [GCaMP6s] (Figures 4, 5). This approach was essential for choosing the best construct for the most challenging recordings, those of the CF spikes *in vivo*. Furthermore, while outside of the scope of the present work, the correlating features of calcium transients in spikes and STOs with the probe concentration is likely to provide useful insights for simulation work elucidating the intracellular calcium dynamics underlying IO physiology. (Negrello et al., 2019).

Fluorescence imaging data is commonly only presented in baseline-normalized form (DFF Macleod, 2012), but when examining a novel probe it is necessary to compare raw intensity values to understand how its expression level affects neuronal signaling. Notably, we did not subject the fluorescence intensity values to convolutional transforms or template fitting when measuring calcium event waveforms as is commonly done. Background was also not subtracted from the values to avoid introducing biasing Artifacts in lowest-amplitude event waveforms. Instead, to allow comparison between varying experimental conditions and event amplitudes, we present the fluorescence intensities in units of power (fW), estimated based on photo-conversion properties of camera sensors (using a window between dark noise and full electron-saturated pixel). Obviously, future studies may employ advanced mathematical approaches (such as Hoang et al., 2020) to increase precision of desired measurements, but they need to be carefully designed for the question at hand.

The following additional experimental details were designed to improve result reliability:

1. GCaMP6s expression time was kept shorter (exactly 15 days) than what was tested with EGFP to avoid overexpression and signal saturation. However, the expression level reached should be confirmed by each experimenter, e.g., by paying attention to whether a significant labeling of the nucleus is visible (Yang et al., 2018)
2. To optimize the dynamic range of the entire dataset, illumination power was adjusted for each field of view separately.
3. Same frame rate (30 fps) was used for all recordings.
4. Correct targeting of virus injection into the IO was systematically checked *a posteriori* of the live imaging experiment using immersion-fixed slices. Data from mistargeted experiments were excluded from the analysis.

Finally, as introduction of an exogenous calcium-binding protein such as GCamP6s always carries the possibility of perturbing cell physiology—even without overt pathological symptoms—it is advised to pay close attention to the expression levels and to avoid recording signals from neurons that display strong fluorescence at rest.

### 4.2. Effects of the Viral Capsid and Promoter on Transgene Expression

Mesoscale transgene expression strength, cell-type targeting (neurons vs. astrocytes) and density are determined by the viral capsid as well as the promoter. Specifically, the viral capsid will influence the number of exogenous insertions in the host cell genome, and the promoter restricts expression of transgene based on the type and developmental and physiological state of the cell. Interactions between capsid and promoter can result in additional variability in expression profiles (Powell et al., 2020), as also was evident in our work. Specificity for structure and cell type is therefore determined by capsid-promoter combination rather than by promoter alone. Out of the 10 promoters examined with AAV9 capsids, only the Htr5b-clones showed structural preference for IO (Figure 1), and within the boundaries of the IO, 6 of the 10 promoters were more strongly expressed in astrocytes than in neurons (Figure 2). This was somewhat surprising and disappointing since similar promoters have previously been used in the context of transgenic animals to drive IO-specific expression, and highlights the importance of viral vector design details. Comparing the three AAV9-derived capsids (AAV9, AAV.PHP.S, and AAV.PHP.eB), transgene expression under the control of Htr5b(3.7) promoter was the lowest with the PHP.S (Figure 4). However, individual, sparsely-distributed IO neurons were labeled strongly enough to allow calcium imaging (Figure 5), suggesting that such an approach could be useful for applications where sub-cellular compartmental imaging is desired. Intriguingly, changing the viral capsid in combination with CAG promoter affected both structure- and cell type -level selectivity. It was beyond the scope of this study to examine the mechanisms leading to these differences, but we strongly recommend future work to be mindful of such possibilities.

### 4.3. AAV-Based Htr5b-tTA/TRE-GCaMP6s Constructs Open Possibilities for Calcium Imaging-Based Investigation of IO Neurons' Physiology

As shown in Figures 8–12, we succeeded in monitoring IO spikes and STOs using simple wide-field fluorescence imaging on *in-vivo* preparations. It should be noted that perfusing the recorded cell with the internal solution of the electrode will necessarily limit the duration of paired patch-imaging recordings to a few tens of seconds, and therefore preventing correlative analysis of large numbers of spikes within a single cell. However, the sensitivity of the probe allowed rough estimation of *in-vivo* widths of IO spikes that have been proposed to play a determinant role in cerebellar function (Mathy et al., 2009; Najafi et al., 2014; Yang and Lisberger, 2014; Roh et al., 2020 Figure 8). Furthermore, with the high-affinity calcium sensor GCaMP6s, we could simultaneously observe features of STO synchronization (Figures 9–11) with time-locked and quasi-synchronous firing (Figures 7, 9, 11) in both *in-vitro* and *in-vivo*. Importantly, electrophysiological activity of IO neurons recorded concomitantly with GCaMP6s imaging *in-vitro* was entirely within the range of values described previously in literature in terms of STO amplitude, frequency and IO spike width variability. This supports the notion that the probes, within the range of concentrations used in our study, do not induce overt changes in IO physiology and thus their use in, e.g., behavioral experiments could be justified. However, detailed investigations of action potential waveforms should, where possible, be conducted with whole-cell patch-clamp recordings. While we did not specifically investigate other probes than GCamP6s, there is no reason to doubt that the constructs described here could be used as vectors for delivering other probes such genetically-encoded voltage indicators (Peterka et al., 2011).

While wide-field 1-photon imaging does not reach the spatiotemporal resolution of advanced 2-photon imaging systems, we could easily resolve single neurons (Figures 6, 7) even in the case of very close neuron neighbors (Figure 7). While further studies will be needed to carefully disentangle subcellular calcium signaling in IO, including calcium dynamics of the mesh-like dendritic neuropil, we consider the identified vector a breakthrough methodological step forward for examining circuit dynamics of the olivo-cerebellar system and investigating IO network activity *in-vivo*. As large part of IO neurons' function emerges from the calcium-based modulation of STOs and spike widths, lack of a well-characterized fluorescent activity probe has been a major obstacle in research of the olivo-cerebellar system. This shortcoming can be thought to be even more severe in IO than other brain regions, as the strong gap junction coupling among the IO neurons (De Zeeuw et al., 1998; Hoge et al., 2011) makes them function less like individuals and more like a syncytium with activity waves engaging continuous stretches of the network. Thus, examining its function *via* single-neuron recordings is bound to fail in providing a comprehensive view of the key computational mechanisms at play.

### 4.4. Calcium Network Activity in the IO Revealed by *in vitro* and *in vivo* Imaging

The results presented in this work mostly pertain to the methodology for systematic viral vector engineering, but as a side product, we report several physiological observations in the IO neurons. First, even though a more thorough study of IO spike generation mechanisms is needed, the linear relationship between calcium event rise time and the width of electrophysiological IO spike is a measure of the possible roles played by somatic calcium conductances in shaping discharge waveform (and thereby the duration of the complex spike; Mathy et al., 2009; Roh et al., 2020; Bazzigaluppi and De Jeu, 2016; Bazzigaluppi et al., 2012). Second, making use of this relationship, we estimate that every 100 ms increase in calcium event rise time corresponds to around 6 ms longer spike width, and shortest measured calcium events with 200 ms rise times correspond to 4–5 ms spike widths. With this insight, the distribution of calcium event rise times *in-vivo* translates to spike widths of 13.7 ± 0.7 ms with some spikes as long as 21.8 ms. Assuming a frequency of 270 Hz for the IO axon burst-firing (Mathy et al., 2009), such spikes could be expected to contain 4-5 “spikelets.” While the calcium recording from IO axons (CFs) were performed using the AAV-PHP.eB-Htr5b construct that likely can not report on the full range of IO spike dynamics, the CF events were similar (Figure 12C2), providing further support to the notion that intrinsic IO dynamics can influence the shape of cerebellar CFs (Yarden-Rabinowitz and Yarom, 2019).

Finally, the present results are the first report of multi-unit recording of IO activity *in-vivo*, showing that the STOs can be frequency-synchronized across many neighboring neurons in a living animal. While this observation still needs to be confirmed in an awake, behaving animal, it gives support to the notion that STO synchronization may persist in the presence of afferent input in the intact brain.

## 5. Summary

The results presented here demonstrate the importance of detailed attention to viral capsid-and-promoter combinations for optimizing genetic targeting for a specific brain structure. Through a systematic engineering effort, we constructed several new vectors that show high potential for the following applications:

- IO spike detection and width estimation: AAV9-Htr5b(3.7)-tTA/TRE-GCaMP6s mix; note that if spike timing precision is a high priority, the GCaMP6s could be replaced with GCaMP-X (Yang et al., 2018).
- IO STO measurements: AAV.PHP.eB-Htr5b(3.7)-tTA/TRE-GCaMP6s mix.
- sparse labeling of IO cells (e.g., for sub-cellular compartment imaging or morphology): AAV.PHP.S-Htr5b(3.7)-tTA/TRE-GCaMP6s (or EGFP) mix.
- specific targeting of IO astrocytes: AAV9-CAG-EGFP.

Using these vectors we show proof-of-principle recordings of both subthreshold and suprathreshold activity of the IO in *in-vitro* and *in-vivo* using easy-to-use 1-photon imaging, with quality resolution and simple image processing tools.

## Data Availability Statement

Anatomical images were archived on ‘BioImage Archive' repository (EMBL-EBI, Hinxton, Cambridgeshire, CB10 1SD, UK) and available online: https://www.ebi.ac.uk/biostudies/studies/S-BIAD408. Plasmids referenced in Table 1 are available on Addgene: https://www.addgene.org/, further inquiries can be directed to the corresponding author/s.

## Ethics Statement

The animal study was reviewed and approved by Okinawa Institute of Science and Technology Graduate University Institutional Animal Care and Use Committee (IACUC).

## Author Contributions

KD, KK, DG, JW, and MYU designed research. KD, KK, DG, and MYU performed research. KD and MYU analyzed data and wrote this article. All authors contributed to the article and approved the submitted version.

## Funding

The research has been funded by OIST intramural support.

## Conflict of Interest

The authors declare that the research was conducted in the absence of any commercial or financial relationships that could be construed as a potential conflict of interest.

## Publisher's Note

All claims expressed in this article are solely those of the authors and do not necessarily represent those of their affiliated organizations, or those of the publisher, the editors and the reviewers. Any product that may be evaluated in this article, or claim that may be made by its manufacturer, is not guaranteed or endorsed by the publisher.

## Abbreviations

CAG: chicken beta-Actin promoter
CaMKII: Calcium/calmodulin-dependent protein kinase II
CF: climbing fiber
CS: complex spike
DFF: normalized fluorescence responses (signal / background)
EGFP: enhanced green fluorescent protein
GECI: genetically encoded calcium indicator
htr5b: 5-hydroxytryptamine receptor 5B
Igsf9: immunoglobulin superfamily member 9
IO: inferior olive
MAP: mitogen pctivated Protein
NeuN: neuronal nuclei antigen
PCA: principal component analysis
pdx1: immunoglobulin superfamily member 9
PSD: power spectral density
RN: reticular nucleus
ROI: region of interest
STO: sub-threshold oscillation
susd4: sushi domain containing 4.
